# Structural Health Monitoring Using Fibre Optic Acoustic Emission Sensors

**DOI:** 10.3390/s20216369

**Published:** 2020-11-08

**Authors:** James Owen Willberry, Mayorkinos Papaelias, Gerard Franklyn Fernando

**Affiliations:** School of Metallurgy and Materials, University of Birmingham, Birmingham B15 2SE, UK; M.Papaelias@bham.ac.uk (M.P.); g.fernando@bham.ac.uk (G.F.F.)

**Keywords:** acoustic emission, condition monitoring, fibre-optic sensors, non-destructive evaluation, structural health monitoring

## Abstract

Acoustic emission (AE) is widely used for condition monitoring of critical components and structures. Conventional AE techniques employ wideband or resonant piezoelectric sensors to detect elastic stress waves propagating through various types of structural materials, including composites during damage evolution. Recent developments in fibre optic acoustic emission sensors (FOAES) have enabled new ways of detecting and monitoring damage evolution using AE. An optical fibre consists of a core with a high refractive index and a surrounding cladding. The buffer layer and outer jacket both act as protective polymer layers. Glass optical fibres can be used for manufacturing AE sensors of sufficiently small size to enable their embedding into fibre-reinforced polymer composite materials. The embedding process protects the FOAES against environmental stresses prolonging operational lifetime. The immunity of FOAES to electromagnetic interference makes this type of sensor attractive for condition monitoring purposes across a wide range of challenging operational environments. This paper provides an exhaustive review of recent developments on FOAES including their fundamental operational principles and key industrial applications.

## 1. Introduction

Non-destructive evaluation (NDE) is useful for monitoring structural components and machinery in real time or during maintenance [1,2,3,4]. Fibre-reinforced polymer composites (FRPCs) consist of a polymer-based matrix and supporting reinforcement fibres. FRPCs are preferred materials in environments that require low weight but high strength and stiffness such as novel bridge designs [5,6], aircraft components [4,7] and wind turbine blades [8,9]. The complex microstructure of FRPCs requires advanced NDE techniques for the detection and evaluation of crack initiation and propagation under cyclic loading conditions. NDE techniques commonly employed include ultrasonic testing [10], infrared thermography [11], shearography [12], radiography [13] and acoustic emission (AE) [14]. AE relies on the detection of elastic stress waves, or otherwise acoustic waves, generated from a structural component when a crack initiates and propagates under certain loading conditions. AE is a particularly useful monitoring method for crack detection and location in components exposed to fatigue loading conditions [15,16,17]. Elastic stress waves generated during crack initiation and propagation in structural components manufactured from FRPCs [18], metals [19], wood [20] and even road pavements [21], are converted to electric signals using Lead Ziconate Titanate (PZT) piezoelectric ceramic crystals [22]. More recently, optical fibre sensors have gradually become more popular for AE sensing based on various designs including fused-tapered couplers [23,24,25], Mach-Zehnder sensors [26,27], Michaelson interferometers, Fabry-Perot interferometers [28,29] and fibre Bragg gratings (FBGs) [30,31]. The detection of Lamb waves emitted by an initiating or propagating crack allows large structures to be monitored using a small number of sensors. Lamb waves are ultrasonic waves that propagate along plate-like structures with lower levels of attenuation than longitudinal and shear waves. Lamb waves enable the detection and monitoring of growing cracks in structural components [32,33,34].

Crack detection and damage analysis capability using AE has evolved further in recent years. The detection sensitivity to even very tiny levels of crack propagation is a distinct advantage that the AE technique offers over conventional NDE techniques. The accuracy of source detection, source type identification, location and damage level evaluation capability of AE has improved thanks to novel statistical and signal processing analysis techniques [35,36]. Lamb waves can propagate over long distances throughout the thickness of the plate thanks to the lower levels of attenuation. This means that AE sensors can be at a distance from the actual area where damage has occurred and still be capable of detecting and monitoring it as it evolves [37,38].

In this review paper, we discuss the principle of fibre optic sensors and the concept of acoustic emission analysis. Furthermore, the principle of operation for several FOAES are discussed, which also include corresponding recent developments and applications.

Section 2 provides information on the fundamentals of fibre optic AE sensors that includes comparing the advantages FOAES have over piezoelectric AE sensors, the typical operational temperature range of FOS, and also some issues with cladding modes. The principle of acoustic emission is also reviewed where both active and passive sensing are mentioned. Acquisition parameters used during all types of AE analysis are also discussed.

Section 3 introduces fused tapered couplers as fibre optic AE sensors, the power distribution associated with them and the techniques employed during manufacture. Appropriate equations to calculate the throughput and coupled power are demonstrated. Further, an investigation into the relationship between temperature, tensile strain and compressive strain is detailed.

Section 4 describes Mach-Zehnder interferometers displaying appropriate diagrams and equations. MZI interferometers used as popular tools for analysis of many other SHM techniques are mentioned, in addition to AE sensing.

Section 5 discusses Michelson interferometers and demonstrates the similarities with Mach-Zehnder interferometers. These are highlighted in the diagrams and equations, though Michelson interferometers are further explored to relate the sensitivity of acoustic pressure waves to the optical path length of the sensing fibre.

Section 6 evaluates Fabry-Perot interferometers and their popularity as pressure and strain sensors, as well as AE sensors. The relationship between the sensitivity of the device and the Young’s Modulus of a material is also considered. A comparison is made between intrinsic and extrinsic Fabry-Perot interferometers.

Section 7 concludes with fibre Bragg gratings. The advantage of them being made from a single optical fibre is discussed in addition to their response to longitudinal and transverse stress waves. Numerous case studies discuss FBGs being used in a variety of circumstances including AE sensing, high temperature sensing, displacement sensing and also strain monitoring in asphalt concrete.

Table 1 provides a brief overview on the types of sensors that are covered in this review, the monitoring methods and techniques, and their typical uses.

## 2. Fundamentals of FOAES

### 2.1. Principles of FOAES

Piezoelectric AE sensors have historically dominated the AE industrial and research applications market. The piezoelectric crystal in most cases is manufactured using doped PZT ceramic. Piezoelectric sensors can be used as both sensors and actuators thanks to the direct and converse piezoelectric effects that they exhibit. AE as an in-service monitoring technique has been studied extensively and results have been reported by various research groups around the world [39,40,41,42]. Fibre optic sensors (FOS) on the other hand, offer the capability of remote monitoring in real-time for analysing the structural integrity of critical components using embedded devices [43,44].

FOS can be used at higher operational temperatures than most conventional piezoelectric ceramic sensors, which tend to have a maximum operational temperature of 150 °C [45,46]. This is true of FOS manufactured from silica. In some cases where they are manufactured from polymer materials however, the maximum operating temperature tends to be reduced to around 110 °C [47].

Although most FOS are manufactured from silica, polymer optical fibres (POF) are sometimes preferred due to their lower acoustic impedance and reduced Young’s Modulus over conventional techniques [48]. Although they are more robust than silica fibres, the advantages are shadowed by the fact that polymer optical fibres tend to suffer high attenuation over long distances making distributed sensing non-viable.

Since the earliest development of FOS for AE analysis [49,50], it has been clear that their performance is unaffected by electromagnetic interference. Hence, the integration of FOS into environments where electromagnetic radiation is present is of no concern.

**Figure 1:**
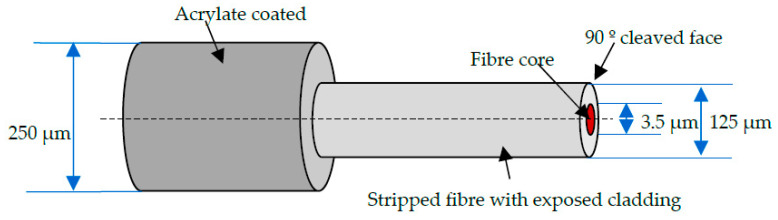
A schematic illustration of an optical fibre used as an alternative to piezoelectric sensors for structural health monitoring.

The majority of piezoelectric sensors are resonant systems operating over a narrow bandwidth of a few tens to a few hundred kHz [51,52]. Nonetheless, there are also wideband AE piezoelectric sensors which offer a flat response over a wider range of frequencies, normally up to 1 MHz. The attenuation of the AE signal with increasing distance is a consideration for all AE measurements. The level of attenuation depends on the material type as well as its geometry. A typical piezoelectric sensor is cylindrical in shape, with a diameter of 20 mm, height of 30 mm, and a weight of around 30 g [53]. Thus, embedding piezoelectric sensors in composite structures is not possible or practical. FOS are lightweight, with a small enough diameter (~250 µm) to enable them to be embedded into composite structures [54]. They can also be multiplexed so as to accommodate multiple sensors simultaneously [55,56,57,58,59]. The typical dimensions of an optical fibre are shown in Figure 1.

The presence of a polyimide/acrylate coating relieves the stress concentration around the cladding during the embedding process of the FOS. Airbus has actively investigated the use of FOS for monitoring components in their aircraft, including the A380 superjumbo [3] and the A340/600 tail wing using FBGs [60]. The data acquired have shown excellent performance in comparison with conventional strain gauges.

Further studies have investigated damage monitoring in carbon-fibre reinforced polymers (CFRPs) by analysing micro-bending [61]. As AE waves generated from a growing crack propagate through a composite structure, they interact with the optical fibre. The displacement caused by the propagating AE wave causes the optical fibre to bend locally. The detected AE waves by the intensity-modulated sensor are analysed for the presence of delamination, matrix cracking and fibre fracture. The FOS AE technique is simple, robust, and able to characterise the frequency content of the detected damage modes. However, its sensitivity has been found to be lower in comparison with interferometric sensors, which include Fabry-Perot, Mach-Zehnder and Michelson interferometers.

Other FOS devices that have been considered as an alternative to piezoelectric AE sensors include fibre optic ring AE sensors (FORAES) [62]. The FORAES design incorporates a sensing frame and a sensing fibre introduced to overcome problems with multi-directional sensitivity. The system is based on the heterodyne interferometric demodulating method, first developed in the 1980s [63]. The system is insensitive to optical power (amplitude) and polarisation fluctuations.

Although praised for their unique capabilities, FOS have certain disadvantages and technical limitations. Their durability during handling and cost are two important disadvantages. Moreover, cladding modes that are observed during or after the fabrication process, adversely influence successful damage detection. These modes permit light to oscillate into the cladding from the fibre core, particularly in single-mode fibres where the light is strongly attenuated into the cladding [64]. Due to different propagation constants, core modes and cladding modes do not noticeably couple with each other. Although the cladding provides a total internal reflection of light propagating through the core, the two modes may interact during a variation of the core/cladding structure. The cladding then becomes the fundamental propagation mode instead of the core [65]. Cladding modes can be more easily scattered and escape the high refractive index core if narrow depressed claddings are utilised, i.e., the addition of a cladding between the existing core and cladding to reduce coupling strength between the guided mode and cladding modes [66,67]. This cladding will often have a refractive index less than any other cladding [68]. For tapered optical fibres, the input light is initially guided through the fundamental mode in the fibre core. The mode orientation brings about changes for the entire fibre, as the original core and cladding are converted to the new core and the surroundings become the new cladding. A smooth tapering permits the transition of light between the core and cladding. Possible solutions to eliminate cladding modes may be to coat the fibre length in an index matching fluid [69].

Little analysis on the costs of manufacture FOAES has been conducted. However, the typical material cost when manufacturing FOAES from the fused tapered coupler method has been approximated to be around five pounds per sensor [70,71]. Another study reported that the material cost of their novel fibre optic ring AE sensor was around two dollars [62]. Although only consisting of an optical fibre and glass capillary housing, as a result of their single-use, FOAES are often very expensive compared with multiple-use piezoelectric AE sensors.

### 2.2. Acoustic Emission

The principle of AE sensing is based on the detection of elastic stress waves or acoustic waves released due to crack initiation or propagation when a structure is mechanically loaded. The characteristics of the waveforms generated are directly related to the source that resulted in them, type of material and component geometry [72]. AE sensing can thus be used for damage source identification and location [73].

The location of damage in structures can be performed either using active [74] or passive means [75]. Active AE sensing refers to activating a material from an external source. The source of information is derived by creating some effect in or on the material by the external application of energy [16]. This method can also enhance unnecessary damage growth within the material.

In passive AE damage, location can be achieved using arrival times of the acoustic waveforms detected by the AE sensors. The piezoelectric sensors subsequently convert the crystal deformations arising from the propagating acoustic waves to electric signals which are thereafter digitised and analysed in order to establish the level of damage sustained as a function of time [16,76]. The major difference between the AE method and other NDT methods is that AE is solely passive.

**Figure 2:**
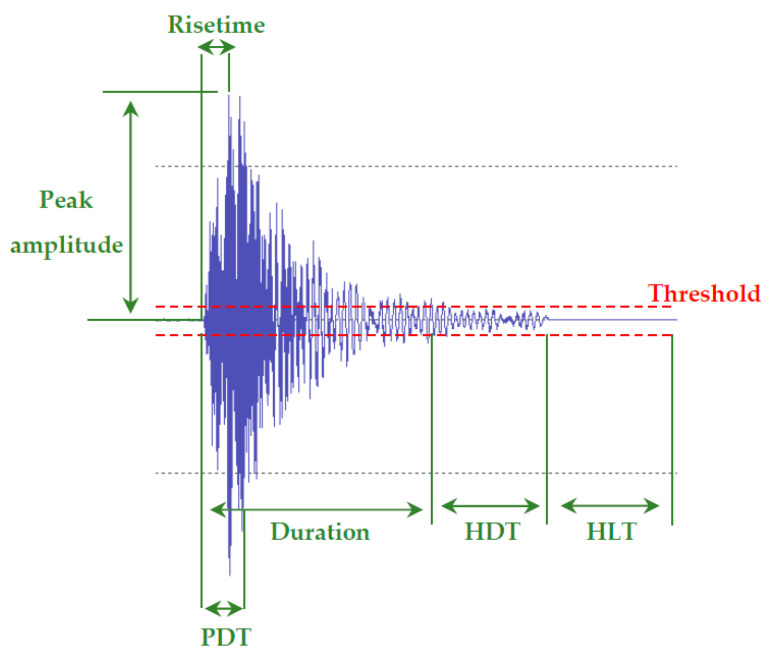
A schematic illustration of an annotated AE signal with appropriate parameters labelled (after [85]).

Effective AE monitoring requires that signals related to damage evolution are successfully separated from unwanted mechanical noise using appropriate filtering techniques. This is possible by amplifying the signal and filtering out the background noise using either appropriate hardware or software-based techniques. Post-processing of the acquired AE events allows the accurate evaluation of the detected damage modes. The main parameters for characterising the signals acquired include, rise time, peak amplitude, energy, duration and frequency content. The amplitude threshold during AE measurements is set so as to eliminate unwanted background noise. An additional parameter considered in damage monitoring of composite materials is the rise time-peak amplitude (RA) ratio. The RA ratio is a useful indicator of classifying damage types in composite structures [77]. The Peak Definition Time (PDT), Hit Definition Time (HDT), Hit Lockout Time (HLT), and maximum signal duration are timing factors that are required to be set according to the nature of the geometry of the material tested so as to enable the accurate detection and evaluation of any damage initiation and subsequent growth under mechanical loading. The Peak Definition Time (PDT) is an acquisition parameter that describes the elapsed time between the signal first exceeding the threshold to the maximum amplitude of a single event. Hit Definition Time (HDT) is the time that an event capture remains open after the signal has passed the threshold. Setting the HDT value low is particularly useful when testing a brittle material with many consecutive AE events. Setting its value too low, however, can adversely influence the maximum peak amplitude of the signal or result in unwanted signals to be acquired. Setting the HDT too high can result instead, in certain damage-related events being missed. The Hit Lock-out Time (HLT) is the time where the AE sensors do not record further signals after an event is recorded [78,79]. The HLT needs to be chosen such that echoes of a primary event are not recorded since they can be mistaken for actual damage events. On the other hand, if HLT is set too high, damage events can be missed since the sensors are not recording during this time. The maximum signal duration is the maximum duration that a recorded AE signal can have. If the recorded signal’s duration exceeds the maximum signal duration value set, then the sensors will stop acquiring and restart acquisition once the HLT has been exceeded. This is a useful parameter for avoiding continuous AE signals arising from background noise to adversely affect the AE measurement. Useful AE parameters are demonstrated in Figure 2.

Simulated reference AE signals using pencil lead breaks or otherwise Hsu-Nielsen source are conventionally used to evaluate the coupling and response of the AE sensors installed in-situ prior to any actual testing. Alternatively, in the place of a Hsu-Nielsen source an impulse generator can be employed instead. The propagating speed of acoustic waves in an aluminum bar were evaluated using AE [80]. The AE signal source was placed at one end, with two AE sensors fixed at either end of the aluminum bar. The time delay in detecting the propagating acoustic waves was used in conjunction with the known distance between the two AE sensors in order to calculate the wave propagation speed. An impulse generator (A DECI, SE25P excited by a Model 600 Pulsar) and a pencil lead-break test were used to generate simulated AE signals. The signals from both AE sources were detected by the second AE sensor ≈1 ms after the first sensor had detected them [31,42,81,82,83,84].

## 3. Fused Tapered Couplers

### 3.1. Principle of Operation

Fibre optic couplers (FOCs) are passive devices used for splitting, multiplexing, and filtering optical sensors. Two fibres are placed into close parallel contact. Their power transfer is subsequently evaluated by assessing their core-to-core distance. For an effective coupling, the core-to-core distance should allow an efficient transfer of light between the fibres with minimal loss. Prior to loading the fibres, stripping of the acrylate coating removes it from the rest of the fibre. Mechanical stripping is one of the cheapest and least time-consuming methods used for this process. After this, the fibres are cleaned to remove any surface impurities which can otherwise adverse scattering losses [86,87]. Fabricating with fibre fusion has the advantage of being a quick and simple technique, unlike other methods including etching [88] and polishing [89]. Fibre fusion has emerged as a cost-effective solution. AE condition monitoring can make use of FOCs fabricated using fibre fusion [90,91]. This is done in conjunction with the fibre bi-conical tapered (FBT) technique [24]. The FBT method subjects two parallel fibres in close contact under a high temperature flame [92] to fuse them. This results in the modification of the fibre-core refractive indices and allows for the light transfer across a tapered region. These intensity-based optical fibre sensors are usually controlled in real-time and emit light via a suitable laser source. The authors from [93] and [94] both made use of a Fabry-Perot pig-tailed benchtop laser source operating at 635 nm with a minimum output power of 2.5 mW. Chen et al. [84], utilised the same laser type to interrogate 2 × 2 optical fibre couplers. Doyle et al. [23], used lasers operating at 633 nm and 670 nm to compare surface mounted and embedded FOCs.

**Figure 3:**
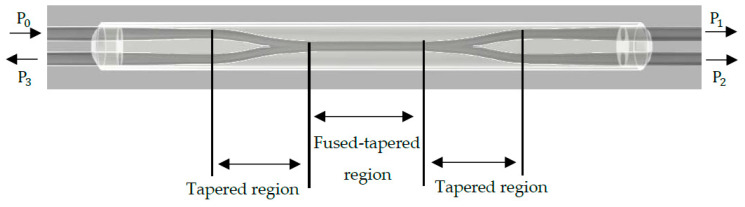
A schematic illustration of a bi-directional 2 × 2 fused tapered coupler. $P_{0}$ represents the light input. $P_{1}$ and $P_{2}$ represent the throughput and coupled power outputs. $P_{3}$ represents the bi-directional (backward/cross-talk) light propagation.

To apply the coupled-mode theory (CMT) to the fused biconical tapered system, a coupling coefficient is introduced [95]. CMT states that the coupling ratio of a fibre coupler changes periodically with the centre distance of two optical fibres [96]. A perfect directional coupler has an input power equal to the power at the outputs $\left(P_{0}=P_{1}+P_{2}\right)$, and has zero backwards reflected power ($P_{3}=\;0)$ with no optical losses. This is shown in Figure 3.

For a single-mode fibre pair, the tapering pulling speed, flame temperature and flame brush width influence the performance of the coupler as a sensor. The pulling signature or distinguishing pulling style, including the pulling force, is affected by these parameters [25,97]. When an acoustic wave propagates across the tapered region, it excites the coupled region and alters the coupling ratio. The coupled region acts as a strain concentrator, magnifying the propagating acoustic wave in this region. This causes a change in the refractive index (RI) and effective length due to the acoustic-optic modulation and mechanical effects, respectively. The RI change is a result of the perturbation of the strain field, whereby it is modulated by an external acoustic wave that changes the coupling ratio between output fibres [98]. The tapering results in an increase in the evanescent field, thus enhancing the interaction of the light with the surrounding medium.

Acoustic energy, optical input power, the coupling region structure and acoustic wave properties contribute to the optical power output of couplers [99]. Over a decade after being first introduced, a fused tapered optical sensor was used to evaluate the performance of the coupler based on the taper symmetry, fibre geometry and measurement tolerance [100]. Glass acoustic-waveguides (such as fused silica) were found to be revealed as advantageous for wave propagation due to their low inherent attenuation of the stress waves in the 20–100 kHz range. The efficiency of the optical coupler is also heavily dependent on the fibre coupling between the fibre pair, known as the coupling ratio *(CR)*. CR is defined as a measure of the power distribution between coupler outputs for a given wavelength range and is expressed as follows [24,101]:

(1) $$\mathrm{Throughput}\;\mathrm{CR}\;(\%)\;CR\;=\;\left[\frac{P_{T}}{P_{T}\;+\;P_{C}}\right]*100\%$$

(2) $$\mathrm{Coupled}\;\mathrm{CR}\;(\%)\;CR\;=\;\left[\frac{P_{C}}{P_{T}\;+\;P_{C}}\right]*100\%$$

Equations (1) and (2) represent the power obtained at the throughput output $\left(P_{1}\right)$ and the coupled output $\left(P_{2}\right)$ for a 2 × 2 coupler.

A system of an identical fibre pair executes a coupling ratio variation of 0 to 100% [99]. Modelling of the relationship between draw length and coupling ratio indicated that a 50:50 splitting ratio between fibres can occur at a draw length of around 11 mm, 13 mm, and 15 mm. Although this is more difficult to control with increasing draw lengths [102]. The coupling variation between fibres continues until fibre fracture occurs under axial strain. The diameter of the tapered region affects sensor sensitivity [103]. A narrower sensing region results in an increased signal-to-noise (SNR) ratio, describing the coupled region as the most sensitive part of the device. The data demonstrates that the effective refractive index of the fibre core is proportional to the taper diameter. This phenomenon has also been observed in fused tapered couplers used for temperature monitoring with taper length, width and taper angle contributing to the sensitivity exhibited by the sensor [104]. A sensitivity increase of 2% was observed when the tapered region was decreased from 12 mm to 4 mm. Due to the diameter-sensitivity relationship, fibre tapers exhibited a SNR of up to 20 dB higher than standard single-mode fibre sensors. It has thus been considered that longer and thinner tapered sections could exhibit larger phase changes to acoustic vibrations. The draw length and coupled power relationship depends on the geometry of the fibre volume at the coupled region, the laser’s wavelength and distance between fibre cores [93]. Furthermore, the sensitivity of a fused tapered optical fibre AE sensor is dependent on the acoustic wavelength. Important functional parameters of these sensors are the sensitivity and broad bandwidth. High sensitivity enables the detection of AE signals with lower amplitude and a broad bandwidth allows the device to be less sensitive to low frequency environmental noise [105].

The relationship between tapered length and waist diameter has been found to be inversely proportional. The relationship has been evaluated when tapering optical fibres during sensor manufacturing [106]. As the tapered length decreases, the fibres are stretched and the tapered region diameter is reduced. Sensors with longer taper regions exhibit higher sensitivity due to their reduced diameters [107]. This is because the strain amplitude of the fibres is dependent on the material properties, acoustic power and the diameter of the tapered coupling region [84].

Excess loss is an important performance parameter in testing couplers. It has been previously reported that couplers with an excess loss of less than 1.0 dB are easily achievable [50]. Those with a loss of less than 0.5 dB were considered to perform satisfactorily, but the best device during sensor evaluation had an excess loss of 0.2 dB and a power splitting ratio of 1:1 between waveguides. The power coupler efficiency relies on the shape and position of the fibres, together with the coupler mode theory. Experiments were conducted to find the relationship between the tensile strain (T_Strain), compressive strain (C_Strain), temperature (Temp) and the coupling ratio of a 2 × 2 fused tapered optical coupler [108], resulting in Equations (3)–(5):

Temp      *R = 0.0336 T + 49.4* (3)

T_Strain      *R = 0.0482 S + 49.7* (4)

C_Strain      *R = −0.0482 S + 49.7* (5)

where, T_Strain and C_Strain are the tensile and compressive strains, respectively. R is the coupling ratio (%), T is temperature (°C) and S is the micro-strain (µℇ).

### 3.2. Development and Applications

The fused-tapered coupler is a well-established component used in optical fibre AE sensing. As the device requires the vibration of optical fibres, the fibre-ends may only be in contact with the substrate, through the sensor packaging. This permits free oscillation of the fibres. For this reason and to offer protection during mechanical loading, FOAES have been manufactured with the coupling waist region fixed in a silica V-groove by bonding one end with UV cured epoxy adhesive and the opposing with silicone rubber. The sensing region itself is left suspended in air [84].

Furthermore, single-mode optical fibres are normally utilised for fused-tapered coupler fabrication due to their greater insensitivity to bending compared with multimode fibres [107]. Another 2 × 2 optical fibre coupler was fabricated from two identical fibres with a 50:50 ratio and placed in a glass rod of 1.5 mm diameter secured with UV cure epoxy. The sensor was coupled to the surface of a unidirectional composite and tested before being embedded during fabrication of the composite panel and re-tested. For embedded sensors, an additional outer glass capillary of 2 mm diameter was secured to the device with UV cure epoxy [109].

A comparison between U-shaped cylindrical and U-shaped square packaging was carried out in order to optimise the device SNR. Improvements to the average signal level of the FOAES were achieved using a smaller-diameter circular U-shaped packaging [94]. Similarly, other FOAES were packaged in silica V-grooves and secured in position using UV epoxy adhesive. A silica tube was then slid over the sensing region of the 2 × 2 coupler and secured in place with the same adhesive [110]. AE couplers were fabricated to examine the power loss across various coupling ratios by twisting/wrapping the fibres around each other. A 90 mm silica capillary with plastic end-faces was placed over the tapered region and secured using epoxy resin. The environmental stability of the devices was determined by manufacturing additional sensors that used a viscous uncured silicone elastomer to fill the capillary packaging. The coupler’s temperature sensitivity was marginally improved due to the thermally induced change in the elastomer refractive index [111].

Fused couplers have become increasingly popular for damage detection in composite materials. Early damage detection is possible during the fabrication and mechanical testing process of the material by embedding the devices between laminates in composites [18]. Furthermore, tests which compared the FOAES with piezoelectric AE sensors on composites were conducted [82,110]. When comparing these two sensor types, the fused tapered coupler is embedded between composite plies whilst the piezoelectric AE sensor is surface-mounted. Upon comparison with an R15 resonant frequency piezoelectric sensor [112], calibration tests proved that the results between devices were comparable with both surface-mounted and embedded couplers. Three-point bending tests were used to load the unidirectional 10-ply carbon fibre/epoxy composite samples. The results of the tests showed that the FOAES was able to detect the acoustic waves generated during damage propagation.

The intensity and energy of the detected acoustic waves can be used to determine the level of damage accumulating in the specimen. Embedded FOAES were also compared with piezoelectric sensors with respect to their capability in determining the AE events arising within the CFRP sample during impact testing [113]. A simple algorithm based on the intersection of hyperbolic curves used four sensors (including two FOAES) to determine the location of an impact event caused by a steel ball drop. The response of the sensors was calibrated and compared before and after embedding the samples. The peak voltage of the signal generated by the embedded FOAES was lower than the surface mounted device, despite its improved coupling. This is often the case however, due to attenuation effects occurring inside the sample when compared with the surface [114]. Furthermore, both the horizontal and vertical velocities detected by FOAES were greater in the case of the embedded sensors when compared with the surface-mounted sensors.

Wind turbine blade certification tests comprise of numerous assessments for static, fatigue and residual strength quantification. However, engineers are often unable to accurately locate audible cracking from the material. As such, static and fatigue measurements in wind turbine blades using piezoelectric AE sensors have become increasingly popular due to their ability to detect, locate and analyse the damage accumulating in the wind turbine blade [115,116,117,118]. Due to the advantages of optical fibre sensing devices, fused tapered couplers have also been used for damage detection in wind turbine blades. One experiment involved the manufacturing of a 2 × 2 fused tapered coupler from two lengths of single-mode 630 nm optical fibre [119]. Sensors were first surface-mounted to a glass-epoxy panel and subsequently excited with simulated AE signals. Their output was compared with that of a commercial piezoelectric AE sensor. The FOS response was 20 dB less in amplitude, which is substantially lower than the response of the piezoelectric AE sensor. Wind turbine blade testing carried out, utilised a single surface-mounted optical fibre sensor. The glass fibre-polyester wind turbine blade tested was monitored until final failure due to fatigue. Eight piezoelectric AE sensors were mounted at various places along the blade for comparative purposes. The number of AE hits recorded by the FOAES was comparable with that detected by the piezoelectric AE sensors. Furthermore, the AE activity detected by the FOAES coincided with the AE activity detected by the piezoelectric AE sensors. However, some FOAES AE data did appear to be slightly delayed in comparison with those detected by the piezoelectric sensors. This was likely due to a slower response time or the difference in the electronics of the acquisition hardware used for the FOAES in comparison with the piezoelectric sensors. Nonetheless, this delay was not observed when embedded sensors were compared with the piezoelectric AE sensors during tensile loading tests. The FOS AE events tended to have longer durations as its output decays more slowly than the piezoelectric sensors. The overall AE data were similar between the FOAES and the piezoelectric sensors. Also, the FOAES were not susceptible to electrical noise associated with crosstalk from other channels.

## 4. Mach-Zehnder Interferometers for AE Sensing

### 4.1. Principle of Operation

**Figure 4:**
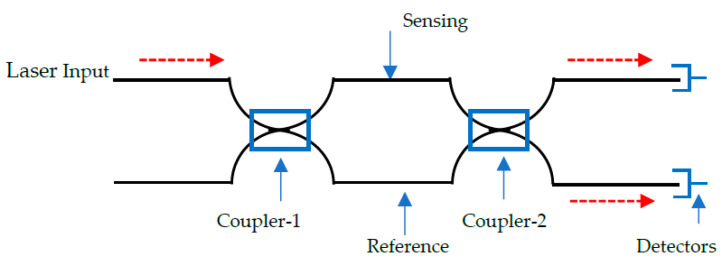
A fibre optic Mach-Zehnder interferometer (MZI) (after [93]).

The optical path of propagating light from a single source is evaluated after splitting it into two components. One beam is defined as the reference parameter, whilst the other (sensing) wave guide responds to the application of mechanical or thermal stresses [120]. A typical setup for this sensor is shown in Figure 4 [93].

A Mach-Zehnder interferometer (MZI) consists of a laser input, beam splitter (coupler-1), two fibres (sensing/reference), beam combiner (coupler-2), and a detector. An external stress presented as a measurand, introduces an effect on the sensing fibre whose light intensity is compared with the reference fibre in a detector. The measurand changes the optical path length of the sensing fibre as the reference fibre remains constant. As such, the phase $\left(\phi_{MZ}\right)$ is a function of the different lengths between the sensing and reference fibre. It can be written as [121]:

(6) $$\phi_{MZ}=\;\frac{N_{eff}\;2\pi }{\lambda_{0}}\;\left(L_{S}-L_{R}\right)$$

where $N_{eff}$ represents the effective refractive index of the fibre, λ is the wavelength of propagating light and $L_{S}$ and $L_{R}$ are the optical path lengths of the sensing and reference fibres, respectively. The variation brought about by the fibre core diameter is negligible and therefore $N_{eff}$ can be replaced by *n*, the refractive index of silica. Moreover, the effective refractive index value is only used when calculating the phase difference in an optical waveguide. Otherwise, this term is ignored when dealing with interfering beams [101].

Mach-Zehnder interferometers are popular tools for analysis of refractive index measurements [122], temperature [123], pressure [124], displacement and strain [120,125]. This study focuses on Mach-Zehnder interferometers for AE analysis.

### 4.2. Development and Applications

Some of the earliest developments in fibre optic AE sensing used a Mach-Zehnder interferometer [49,126]. A variation in the optical path length of the fibres was observed when a fibre was subjected to displacements arising from a propagating acoustic wave. As such, by monitoring the output from the interferometer device, the characteristics of acoustic waves can be evaluated. The MZI has been reported to be able to detect AE events at frequencies ranging from 40 kHz to 400 kHz with negligible sensitivity fluctuations. MZIs can hence be used for analysis of damage modes and failure mechanisms using AE [26,27]. The data has been compared with conventional AE sensing instruments. Furthermore, MZIs have been designed to evaluate damage in composites, with particular attention focusing on delamination in beams manufactured from FRPCs [127]. The signals acquired with the piezoelectric sensors indicated that wave attenuation is higher at higher frequencies. The study proposed Equation (7) for determining the dominant frequency in a composite beam. The speed of the waves propagating through the specimen was determined using two piezoelectric sensors. Thus, the dominant frequency between the top and bottom surface of the beam can be given. The depth of the beam (*D*) and wave speed $\left(C_{P}\right)$ calculated the dominant frequency (*f*):

(7) $$f\;=\;\frac{C_{P}}{2D}$$

The sensitivity of fibre optic AE devices has been investigated using Mach-Zehnder interferometers. Phase shifts in optical fibre have measured pressure-induced phase shifts by phase modulation detection [128,129]. An enhancement of the sensitivity to elastic stress waves was observed by embedding the sensors into a composite structure of low elastic modulus. A signal enhancement of between 10 and 100 times that of a bare fibre was observed. Based on the same technique, another sensing fibre with a polymer coating layer was tested, exhibiting an enhanced detection sensitivity to acoustic waves. A material of low-bulk modulus and high Young’s modulus was preferred for the coating [130].

Mach-Zehnder interferometers have been used to determine the location of AE sources in a structure [131]. The location of AE events was determined by measuring the time delay between two MZIs. Both the sensing and reference arms were 20 km in length and were designed to be applicable for large structural health monitoring with a long sensing range. The simplicity of multiplexing optical fibre sensors was demonstrated when numerous sensing arms shared the same reference arm, light source and photodetector for an amplitude-multiplexed acoustic sensor array [132]. The modified MZI system enabled the development of a cost-effective measurement system with a high level of location detection accuracy. An array of two sensors demonstrated a location accuracy of better than 3 cm.

An alternative solution to AE monitoring came with a membrane-free MZI method that was developed to detect sound pressure induced changes of the refractive index of air in an open cavity [133]. The technique enabled a flat frequency response over a broad bandwidth of 500 Hz to 200 kHz. A cover was added over the air cavity to cut off the modulation of the refractive index, which enabled the estimation of the influence of the vibration in the system. However, a decrease in sensitivity was observed across the entire frequency range when this occurred.

## 5. Michaelson Interferometer for AE Sensing

### 5.1. Principle of Operation

**Figure 5:**
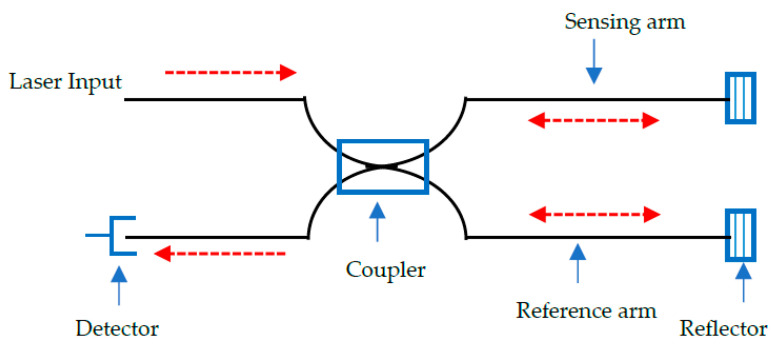
A fibre optic Michelson interferometer (after [93]).

In a similar way as MZI, the Michaelson interferometer (MI) splits a laser beam to detect and identify acoustic waves propagating through structural materials, including FRPCs. A directional coupler splits the propagating light into equal components along the sensing and reference arms. The sensitivity of the interferometer is dependent on the length of the exposed fibre. Thus, the two arms adopt the same length [27]. At the end of fibres waves are reflected, transmitted back through the directional coupler and into a detector [134]. A schematic illustration of this sensing system is shown in Figure 5 [93]. The transmitted power leaves the interferometer at the same edge of the coupler as the input. The light transmission curve for a MI adopts a cosine function to determine the transmitted power $\left(P_{T}\right)$ [135]:

(8) $$P_{T}=P_{o}{cos}^{2}\left(\frac{\phi }{2}\right)$$

with:

(9) $$\phi_{MI}=\;\frac{N_{eff}\;2\pi }{\lambda_{0}}\;\left(L_{S}-L_{R}\right)$$

where the phase difference is identical to that described by the Mach-Zehnder interferometer in Equation (6).

As MIs and MZIs both rely on a directional coupler with a reference and sensing fibre, both interferometers follow a cosine squared function and subsequently the same equation for calculating the phase difference.

### 5.2. Development and Applications

Michelson interferometers have grown in popularity in recent years due to their ability to detect damage and monitor damage evolution mechanisms in composite materials. A sensing arm from a 2 × 2 coupler was mounted to unidirectional carbon fibre/epoxy composites. The response to dynamic and static loading was investigated. Furthermore, the sensing fibre was fitted with a mirrored end to increase reflectivity. The reference fibre was held in a strain-free state. The surface mounted MI could monitor AE events in real-time and detect an axial strain of up to 1.5% [136]. The collected AE data were comparable with those acquired with piezoelectric sensors. In other experiments, the propagation of damage modes within composite materials have been analysed with alternative methods. It is possible to use Michelson interferometers to compare composites as the two fibres are embedded into materials of different layup/matrix conditions [137].

Lamb wave measurements in aluminium, carbon fibre and glass fibre reinforced composites have been evaluated by an MI [138]. A 2 × 2 bi-directional coupler monitored the out-of-plane surface displacements associated with propagating Lamb waves. The sensing system was able to clearly resolve the independent arrival times of the symmetric and anti-symmetric modes.

A different application of a Michelson interferometer was developed in order to sense AE events generated from the partial discharge of high-voltage cable accessories [139]. A single-mode fibre was used due to its compatibility with various instruments and its mass production. The sensing arm of the device was wrapped around a cylindrical elastomer that enabled a broad frequency detection range, whose connection between sensing region and elastomer allowed for a high sensitivity [27]. This was represented in Equations (10)–(12). As the acoustic pressure waves change the optical path length of the sensing fibre, the phase (ϕ) of the sensing fibre was given by a modified version of Equation (9) [139]:

(10) $$\phi \;=\;\frac{2\pi n_{eff}}{\lambda_{0}}\;L\;\left(t\right)\;=\;\beta L\;\left(t\right)$$

where the phase, which is a function of the different lengths between the sensing and reference fibres, is equal to the sum of the propagation constant (β) and optical fibre length (*L*).

As the amount of change to the optical fibre length is governed by the AE pressure waves, the following is true [139]:

(11) $$\Delta \phi \;=\;\beta \Delta L\;\left(t\right)+\Delta \beta L\;\left(t\right)\;=\;\Delta \phi_{1}+\Delta \phi_{2}$$

$\Delta \phi_{2}$ relates to the effective change in fibre diameter, which was proven to be negligible in the article. An equation representing the phase ($\Delta \phi_{1}$), which refers to the axial stretching of the optical fibre was given [139]:

(12) $$\Delta \phi_{1}=\;-\frac{\beta L\;\left(t\right)}{E}\left(1-2v\right)\;\Delta P$$

The phase ($\Delta \phi_{1}$) is related by the propagation constant (β), the optical fibre length (*L*), the Young’s Modulus (*E*), the Poisson ratio (*v*) and the acoustic pressure change (ΔP).

According to Equation (12), the axial stretching of the fibre is executed as an acoustic pressure wave interacts with the optical fibre, changing the optical path length at the sensing region. The change in propagation constant depends on the change in effective refractive index, which in-turn is an effect of the elastic-optic effect and the fibre diameter, produced by strain.

In the study [139], the MI sensing fibre was wound around a cylindrical elastomer to promote a high sensitivity to acoustic pressure changes, which were applied through the cylinder during testing. The fibre length changes were increased due to the mechanical deformation of the elastomer from its larger Poisson ratio. As such, materials with a large Poisson ratio equates to a high sensitivity to acoustic pressure changes.

## 6. Fabry-Perot Interferometers for AE Sensing

### 6.1. Principle of Operation

The Fabry-Perot interferometer (FPI) successfully utilises a Fabry-Perot cavity as an optical fibre sensor that enables the detection of numerous parameters including pressure [140], strain [56] and acoustic emission [141]. The FP cavity is made up of two parallel, highly reflective surfaces divided by a set distance [142]. In a strain sensor, the strain applied through axial loading to the fibre sensor microscopically alters the cavity gap. A laser source is generally utilised to excite the fibres, whose wavelength constructively and destructively interferes with the highly reflective fibre-ends. As FPIs consist of two highly reflecting mirrors, they often incorporate a variety of techniques to minimise the reflection loss and maximise the fringe visibility of the devices. Although FPIs cannot be serially multiplexed due to their inherent nature, they do have static and dynamic measurements capabilities [143].

A low-cost fibre Fabry Perot Interferometer (FFPI) was developed [144] based on two closely spaced ultra-short Bragg gratings within a fibre core. Problems that arise in FFPI designs include their inability to achieve low optical power loss and high coupling ratio within the sensor cavity. These issues were addressed with the presence of a low reflectivity and insertion loss. A low insertion loss allowed for large sensor arrays along a single fibre with minimal signal loss. Short Bragg grating lengths significantly reduced the optical power loss of each FFPI, considerably increasing the multiplexing capability of these devices.

**Figure 6:**
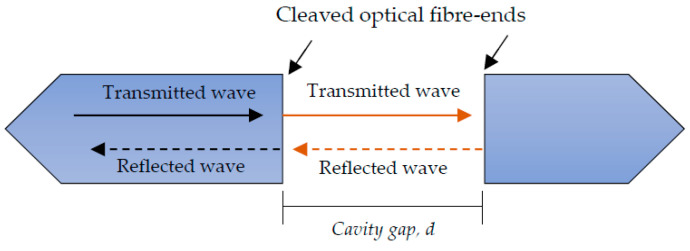
The principle of a Fabry-Perot sensor.

Furthermore, fibre Fabry-Perot sensors require the cladding/core to be accessed during manufacturing, as an optically flat fibre surface is preferred for maximum surface reflection. Although mechanical stripping is preferred for its speed, wet chemical etching is a reliable, cost-effective and simple alternative to mechanical etching during manufacture [88]. A micro-gap was created at a fibre end by a fast etching rate at the core, compared with the cladding. The miniature size of the gap was crucial for minimising transmission loses from the cavity. After etching, two fibres were spliced together with a short arc time and low arc power, which was preferred to avoid over-melting of the micro-gap [145]. Fibre Fabry-Perot sensors for AE consider local elastic stress waves inducing strain in the sensor. As such, acoustic waves periodically alter the dimensions of the FP cavity and cause the reflected light intensity to be modulated. This modulation amplitude usually depends on the optical wavelength and cavity length [146]. A schematic illustration of the Fabry-Perot sensing principle is displayed in Figure 6.

### 6.2. Development and Applications

One of the earliest experiments based on a Fabry-Perot interferometer was carried out in 1978 [147]. In this experiment, one single-mode optical fibre was used as a sensitive AE sensor. This was employed with a high coherence length source by splicing the fibre ends perpendicular to the axis. Thus, the back-reflected optical beams at the sensing terminal would remain excited in the single propagating mode. Unfortunately, there were random amplitude variations during tests, which were due to low frequency acoustic noise, vibrations, and other effects. Coating the fibre ends was considered to increase the reflection coefficient and the resulting signal amplitude.

Nowadays, FPIs are often used as conventional refractive index sensors that employ phase modulation interferences responding to changes in the refractive index of a medium as it fills a monitored cavity. The reliability of the signal is adversely affected by contaminants based on this approach. This means that the fibre surface treatment is crucial. However, this can be minimised by using an FFPI for AE detection. One system designed for AE detection in CFRPs [146], used a Fabry-Perot cavity manufactured from two single-mode fibre ends that were then inserted into a steel capillary to ensure a highly accurate angular alignment. Gluing fibres into the capillary was found to introduce creep during tensile loading. Surface-mounted and embedded devices were compared. A typical sensitivity frequency range between 135 kHz and 215 kHz was observed. Furthermore, pencil lead break tests compared the directionality of the device showing that the highest sensitivity was at the 0° angle from the sensor axis and the lowest sensitivity at the 90° angle.

A surface AE wave sensor based on an in-line extrinsic Fabry-Perot interferometer was demonstrated in the early 1990s [148]. A single-mode fibre was fused inside a 1 cm long hollow core fibre with reflections detected from a multimode fibre. For maximum sensitivity, a cavity gap of less than 1 µm was required. The sensor was placed on an aluminium block and its response to AE waves compared with a piezoelectric sensor. During testing, one end of the fibre was spliced to an EFPI sensing head and the other soaked in an indexing matching gel to prevent false reflections. Low frequency impact acoustic waves and high frequency continuous acoustic waves were detected using this sensor.

In the mid-1990s [149], an alternate FP AE sensor was developed, which consisted of an extrinsic optical fibre sensor utilising a thin polymer film as a low-finesse Fabry-Perot interferometer. The aim was to increase the sensitivity and decrease the susceptibility to unwanted environmental noise. It was recognised that if the phase of the ultrasound field over the sensing region was not the same along the fibre, the peak pressure of the field may be underestimated. As such, an extrinsic Fabry-Perot optical fibre sensor based on the detection of acoustic stress waves by monitoring the thickness of a thin polymer film, was initiated. The system was based on a low-finesse FPI and was mounted to the end of an optical fibre. As the polymer film has a short path length, it exhibits a low exposure to environmental thermal and pressure fluctuations. Hence, complex polarisation systems are academic.

The theory behind the system was supported with the evidence that a film thickness of a few tens of micrometres, provides a megahertz bandwidth. The low Young’s modulus of polymers compared to fused silica also provided a greater sensitivity. The sensing region consisted of a multimode optical fibre secured inside a sensor head, which contained a transparent polymer sensing film with a water-filled cavity gap. The water increased the reflection coefficient providing optimum fringe visibility and delivered an acoustic-impedance match on the fibre side of the film. The strain provided by an incident acoustic wave produced a change in thickness of the polymer film (dl), promoting a phase shift (dϕ). Ignoring strain-induced changes in the refractive index, the following equation can be written [149]:

(13) $$d\phi \;=\;\frac{4\pi \;n\;dl}{\lambda }$$

where *n* is the polymer film refractive index and λ is the wavelength of the laser source. The change in film thickness was given by the following equation [149]:

(14) $$dl\;=\;\overset{1}{\underset{0}{\int }}\frac{P_{T}\left(x,t\right)}{E}\;dx$$

where *E* is the Young’s modulus of the polymer film and $P_{T}\left(x,t\right)$ is the spatial distribution of the pressure across the sensing film thickness. It is the sum of the transmitted acoustic wave and the subsequent reflection.

The new sensor permitted the detection of acoustic waves with a high sensitivity, wideband frequency response. Furthermore, it was demonstrated that intrinsic sensors can detect acoustic waves at ultrasonic frequencies but have a lower sensitivity in comparison with conventional piezoelectric sensors. Similarly, another study [148], concluded that intrinsic devices suffer from complicated procedures and are susceptible to extraneous perturbations. As both [148,149] demonstrated, the sensitivity of an extrinsic Fabry-Perot interferometer (EFPI) to AE, continuously varies if the sensor is under external quasi-static change. As such, it is easy to detect AE in the most sensitive range, though difficult in the least sensitive range. They can operate in a stabilised manner over long periods. Their uniform frequency response is also improved over intrinsic sensors because of the absence of the acoustic-impedance mismatch between fused silica of the optical fibre and the air/water of the cavity gap. Thus, there have been numerous recent studies that consider the use of extrinsic Fabry-Perot interferometers.

One such study reported on an EFPI to detect damage modes in composite materials to compete with other optical fibre damage detection instruments such as fused-tapered couplers and fibre Bragg gratings. The need to reduce the influence of the fade-out effect was highlighted in the study [150]. A phase compensating technique was identified as a necessity when reducing the fade-out effect. An interrogation system that minimised the fade-out effect and showed the AE signals generated from evolving damage during tensile loading a cross-ply composite. The sensor was bonded inside a silica capillary tube and each fibre end-face gold sputter coated. The sensor detected multiple AE events with a crosshead speed of 0.1 mm/min. Failure occurred at 1.6% strain, with cracking initiating at 0.39% strain. The rate of AE events between 0.9% and 1.14% strain was higher than any other damage region, possibly due to matrix cracking and delamination. Above 1.14% strain, fibre cracking was identified as the main damage source. More recently, a fibre optic AE sensor based on a FPI was demonstrated to successfully detect AE events whilst experiencing large quasi-static strains [151]. The system was based on a chirped fibre-Bragg grating with a smart feedback control used to detect AE waves under low-frequency and large background strain. Other studies have considered FBGs to manufacture FPI devices for AE sensing [28,152].

## 7. AE Fibre Bragg Gratings

### 7.1. Principle of Operation

**Figure 7:**
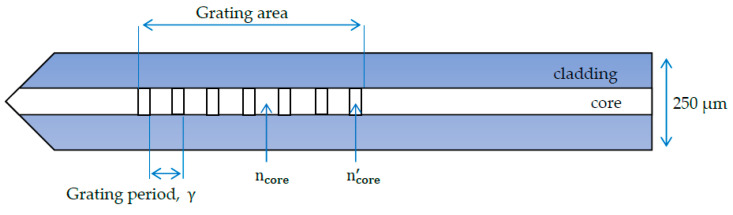
A schematic illustration showing the effect of the refractive index after engraving a fibre Bragg grating onto an optical fibre.

**Figure 8:**
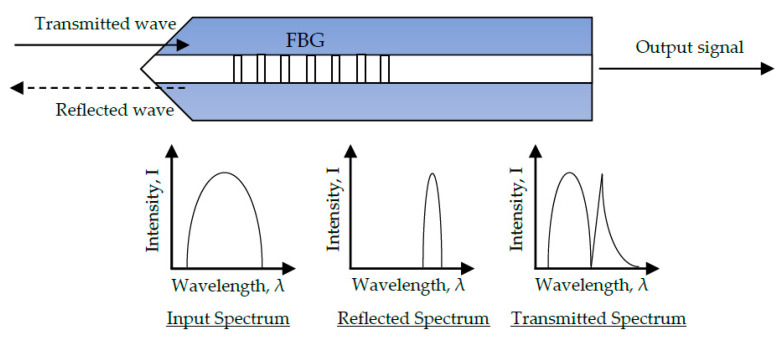
Typical transmission and reflection spectra from a fibre Bragg grating sensor (after [154]).

The conventional fibre Bragg grating (FBG) is made by inscribing a grating into the core of an optical fibre via a high-powered laser. A germanium-doped silica fibre core is exposed to ultraviolet light radiation, leading to a non-reversible effect that creates periodic changes in the refractive index of the fibre core [101]. Light passes through the grating at a specified Bragg peak wavelength, where it is amplified and detected by the fibre sensor. Since the wavelength of this reflected signal is proportional to the grating period, axial strain can be measured through a wavelength peak shift. The Bragg peak wavelength is expressed as [153]:

(15) $$\lambda \;=\;2\gamma n_{eff}$$

(16) $$n_{eff}\;=\;\frac{n_{core}+n_{core}^{\prime }}{2}$$

where, λ is the Bragg peak wavelength, γ is the grating period, $n_{eff}$ is the effective refractive index of the fibre core. The parameter $n_{core}$ is the original refractive index of the fibre core and $n\prime_{core}$ is the refractive index of grating region. It is often the case that $n_{core}^{\prime }>n_{core}$. Schematic illustrations of a typical fibre Bragg grating are given in Figure 7 and Figure 8.

According to Equation (15), the shift in the Bragg wavelength is positive when the FBG expands and is negative when the sensor contracts. In temperature sensing, the fibre length change associated with temperature in silica fibres, only accounts for around 10% of the overall change. The majority of the effect is dominated by a refractive index change [155]. Despite this, these sensors have also been utilised for AE monitoring more extensively in recent years.

It is often the case that the FBG cladding is etched to form a tapered shape so as to enable AE coupling [156,157,158], which ultimately allows a more narrow-band grating reflectivity. Moreover, the acoustic amplitude is increased together with sensitivity.

Lamb waves propagate in parallel to the surface of a structure made of thin plates. When the FBG is exposed to these parallel-propagating waves, the grating pitch on the sensor causes a Bragg wavelength shift. By detecting this shift it is able to reconstruct Lamb waves [159]. Hence, the elastic stress waves generated, source location, damage size and growth can be detected. Upon manufacturing of the FBGs for AE monitoring, the grating length should be less than the wavelength of the Lamb wave [160]. With grating lengths longer than the wavelength, the grating response is insensitive to the acoustic waves generated since they are undetectable. Analysis of the ratio between these lengths was described. A key parameter affecting the grating’s response to Lamb waves is described by the ratio between the ultrasonic wavelength and grating length. Furthermore, for wavelengths approaching the grating length, the main peak amplitude undergoes modulation. At very high ultrasonic frequencies and very low wavelengths, the grating is no longer sensitive.

Analysis of the response from FBGs to longitudinal and transverse waves has been further discussed in other studies [160,161]. The response of a longitudinal ultrasonic stress wave is based on the geometric and elasto-optic effect. Such that a refractive index modulation occurs due to the mechanical contribution of the wave and due to the elasto-optic effect. The effect is represented by the Bragg effective refractive index modulation under the ultrasonic wave equation [160]:

(17) $$n_{eff}^{\prime }\;\left(z^{\prime },t\right)\;=\;n_{eff\;0}-\Delta n{sin}^{2}\left\{\frac{\pi }{{Ʌ}_{0}}z\right\}-\left(\frac{n_{eff\;0}^{3}}{2}\right).\;\left[P_{12}-\;v\left(P_{11}+P_{12}\right)\right]\;.\;{ɛ}_{m}cos\left(\frac{2\pi }{\lambda_{s}}z^{\prime }-\omega_{s}t\right)$$

where, $n_{eff}^{\prime }$ is the effective refractive index of the Bragg grating under a longitudinal ultrasonic wave (LUW). $P_{11}$ and $P_{12}$ represent the stress-optic coefficients/tensor elements, whilst *v* is the Poisson’s ratio. For an elasto-optic interaction with silica fibres. ${Ʌ}_{0}$ is the grating period, $n_{eff}{}_{0}$ is the unperturbed effective refractive index and, Δn equals the maximum index change. ${ɛ}_{m}$ is the LUW displacement amplitude normalised to the LUV wavenumber $2\pi /\lambda_{s}$. $\omega_{s}$ is angular frequency and $\lambda_{s}$ the acoustic wavelength corresponding to that propagating in the host structure. $z^{\prime }$ represents the mechanical deformation on the *z*-axis of the grating under the LUW, at point ‘z’ along that axis.

Transverse waves are significantly more difficult to represent multimode coupled equations and have been modified to support the development of a theoretical model for a uniform FBG subjected to a transverse wave. When this occurs, the multimode coupling equations are modified and adhered to, as detailed in [161,162].

FBGs can be used as reliable, in-situ sensors capable of non-destructively monitoring, and diagnosing the condition of mechanically loaded structures with simple or complex geometries. They possess an excellent resolution and range. A drawback, however, is the effect of birefringence on surface-mounting and embedded configurations. It has been reported that birefringence can lead to different results from mechanical applications. A surface mounted FBG was found to exhibit a sensitivity loss of 42% though a low birefringence maintained the unique Bragg condition. Similarly, an FBG embedded into a composite demonstrated that the cure residual stress could break down the unique Bragg condition [163]. As such, the FBG becomes a transverse sensitive grating. Despite this, FBGs have become capable of being insensitive to environmental perturbation arising in various industrial or environmental applications [153,164]. This was further examined with Mach-Zehnder interferometers made up of long period Bragg gratings [165]. FBGs represent an attractive means for obtaining the vibrational response of a structure. The major advantages of this technique are its compatibility with CFRPs, high resistance to fatigue and excellent multiplexing ability [30,166]. Their very low insertion loss ensures their suitability to multiplexing, in scenarios where time and wavelength division multiplexing may be applied [154].

### 7.2. Development and Applications

There have been numerous studies of AE FBG sensors in recent decades. The first successful fabrication of a fibre-core Bragg grating from a coherent two-beam UV interference pattern on silica fibres dates back to the 1970s [167]. Over the years, the development of the sensing capabilities has evolved to embed optical fibres into composite laminates. Embedding of FOS has become viable with limited impact on the structural integrity of the host material [168]. As such, FBGs have been embedded into concrete composites for traffic load monitoring and assessment of bridges and buildings [169,170]. However, embedding FBGs for macrostrain monitoring poses various challenges, including peak splitting under lateral loading. Known as birefringence, fibre damage can arise as a direct effect of unequal loading. As such, a fibre protective coating was proposed based on a material with low elastic modulus and high Poisson ratio. The protective coating can transfer stress from one direction to the mutually perpendicular directions, solving the lateral loading problem [171]. Silicone rubber was preferred for this coating due to its durability and high-range temperature stability of −100 °C to 320 °C.

FBGs can be employed for the identification of damage modes using AE analysis. One study investigating FBGs evaluated the detection of delamination in fibre reinforced composites [30]. Another relevant study used FBGs to look into for the evaluation of damage evolution in an aluminium plate using AE [31]. The sensing system consisted of a piezoelectric pulse emitter, coupling media, aluminium plate and FBG receiver. Using a strain isolated temperature-only FBG sensor further improvements to enable detection of acoustic waves were made. Moreover, a novel method of identifying damage sources in carbon fibre reinforced polymer composites was developed, based on highly sensitive phase-shifted FBG sensors [172].

The characteristics of Lamb waves were examined with strain-insensitive fibre from micro cracking in the laminates during tensile loading. The amplitude of the symmetric $\left(S_{0}\right)$ mode was found to be dominant during delamination and fibre fracture. The amplitude of the antisymmetric $\left(A_{0}\right)$ mode was found to be dominant for matrix cracking. The FFT analysis of the acquired signals revealed that the peaks observed in the range of 180–390 kHz were related to transverse cracking, whilst the peaks observed in the range of 410–900 kHz were related to delamination, and in the range of 750–900 kHz were related to fibre fracture.

**Figure 9:**
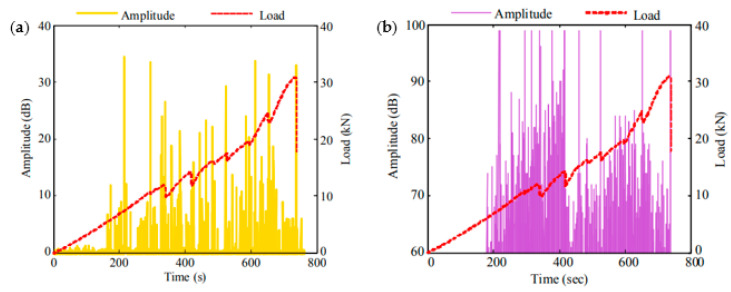
Amplitude verses time measured by the: (**a**) phase-shifted FBG sensor and; (**b**) piezoelectric sensor [173].

Tensile loading tests were conducted on another phase-shifted FBG sensor for the continuous and in-situ monitoring of E-glass/Vinylester top hat stiffeners, normally used in high performance yachts [173]. The amplitude measurement correlated well between the FOS trace and piezoelectric sensor with both devices exhibiting high-amplitude peaks at the time of main failure modes occurring. The FOS amplitude trace was also synchronized with the load changes (Figure 9). The FOS was highly sensitive to small perturbations observed in the detected AE events. The frequency response did not so finely match the response of the piezoelectric sensors, and thus could be improved by refining the sensor acquisition rate, coupling media and stability during operation.

Two FBG sensing systems were manufactured to evaluate the response of the sensor to ultrasonic waves, both of which used different light sources: a broadband light source and a tuneable laser source. The systems were used for the ultrasonic inspection of damaged cross-ply CFRP samples and compared with piezoelectric sensors. An ultrasonic transmitter generated ultrasonic waves propagating through both damaged and healthy regions of the samples tested [174]. The ultrasonic source solely transmitted shear waves through the composite with a peak frequency of 250 kHz. The responses of FBGs and a piezoelectric reference sensor were compared. Symmetric mode waves travel faster through 0° layers within the damaged area than in the healthy area since waves travel faster in materials with a higher Young’s modulus. FBGs have been found to be more sensitive to symmetric modes over anti-symmetric modes [175]. The wave velocity recorded by the surface mounted FBG on the CFRP sample agreed well with the result obtained with the piezoelectric reference sensor. Moreover, the piezoelectric reference sensor was unable to distinguish symmetric mode waves of the damage area from the intact area. The FBG could detect the presence of symmetric mode waves propagating within the specimen. However, the response to antisymmetric modes could not be determined.

Damage detection in CFRPs with FBGs was further evaluated with small-diameter optical fibre FBG devices [176]. With an outer diameter of 52 µm, the sensors resulted in minimal FRPC strength reduction when embedded in parallel to the fibre reinforcement. FBGs are sensitive to non-uniform strain distribution along the grating length, and as such, results in a deformation of the reflection spectrum from the sensor. The relationship between gauge length of the FBG and wavelength of the interacting stress wave is an important concept, since the FBGs are also sensitive to non-uniform strain in the grating. As such, simulations carried out showed that a gauge length of 1/7 of the ultrasonic wavelength was required for optimum FBG detection capability of acoustic waves propagating in the structure. It was also observed that the sensitivity of the FBG depends on the propagation direction as the maximum amplitude of received waves could be expressed by a cosine function of the propagation angle. The occurrence of delamination was easily detected by the small-diameter FBGs due to a decrease in maximum amplitude when a new wave was received. Moreover, the delamination length could be determined from the ratio of the amplitude and Time of Arrival (TOA) of the new mode. Thus, Lamb wave detection was successfully achieved using small-diameter FBGs enabling the effective detection of damage in composites.

FBGs have been directly utilised for strain monitoring on pavement structures [177]. Vertical and transverse sensors were manufactured to allow 3D strain sensing. The sensor dimensions were optimised according to the host structure characteristics. Compressive stress and strain transfer error were minimized by applying an end-annulus at the end of the sensor assembly. The system was tested by monitoring the 3D strain of various asphalt concrete pavement layers of a highway system under different static loading conditions. Upon comparison of in-situ and simulation results, the proposed FBG system successfully and effectively monitored the strain of the highway pavement system.

**Figure 10:**
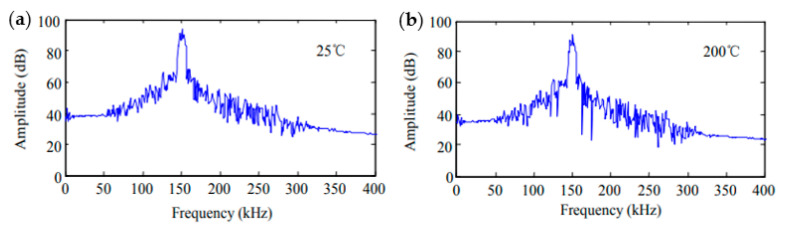
The frequency response of the novel high temperature fibre Bragg grating across the evaluated temperature range at: (**a**) 25 °C and; (**b**) 200 °C [45].

An important advantage of FOS over conventional piezoelectric sensors is their high temperature resistance. Temperature resistant FBGs for AE sensing have been of interest to the industry for a variety of applications. An FBG sensor was manufactured from high temperature fibre and with an operational temperature range of −50 °C to 400 °C [45]. Experiments conducted showed that the resonant frequency increased with decreasing sensing length but was not affected by large temperature fluctuations. Furthermore, the amplitude of the signal remained stable throughout the tests (Figure 10). 

**Figure 11:**
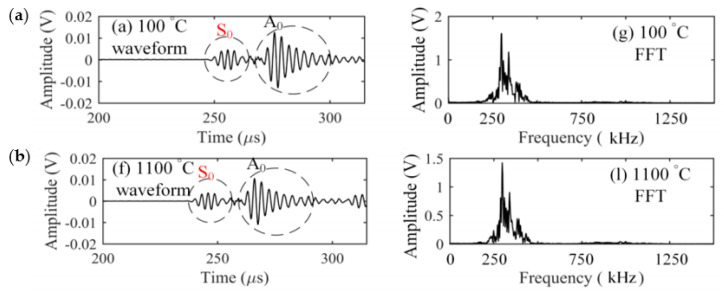
A demonstration of the response of a high temperature FBG sensor to an ultrasonic wave of central frequency 300 kHz, at: (**a**) 100 °C and; (**b**) 1100 °C [46].

Another fibre optic FBG sensor was manufactured using high temperature resistant silica glass fibres. The device was used to successfully carry out AE measurements at over 1000 °C (Figure 11) [46]. Upon comparison of the detection of capability with high-temperature piezoelectric sensors, it was seen that the FBG sensors attained a significantly higher sensitivity over a broad frequency bandwidth. Moreover, at high temperatures the sensor demonstrated an excellent stability response over an 8 h duration.

FBG sensors and conventional piezoelectric sensors were compared in terms of their damage detection capability in structures using AE [178]. Two driving frequencies (260 kHz and 460 kHz) were selected to generate fundamental AE waveforms. The higher frequency gave better results due to the reduced susceptibility to interference of the propagating acoustic waves with acoustic waves arising from background noise sources. Furthermore, the ratio of the damage-generated waves against wavelength size are bigger, which results in a higher SNR. Simulations were conducted to evaluate the influence of the ratio of the fibre grating length against the AE wavelength. The simulations were experimentally verified, and the results predicted a large decrease in modulation depth when the ratio decreases. The results could be used to calculate the maximum allowable ultrasonic frequency for Lamb wave inspection for an FBG of a given grating length. This could also be replicated to find the maximum grating length for a given ultrasonic frequency. Finally, it was shown that the sensitivity from both the FBG and piezoelectric sensors were comparable and had the same SNR. The FBG response to AE waves is dependent on the relative positions of the signal source against the FOS. Thus, the FBGs possess a high directionality.

FBGs have also been employed for AE evaluation of carbon fibre reinforced plastic tanks. A strain insensitive FBG was mounted to a solid rocket motor case to monitor AE signals when the tank was filled with water [179]. A conventional wideband piezoelectric sensor was also mounted as a reference sensor. Both sensors detected AE events when the applied pressure exceeded 1 MPa, continuing up to the maximum pressure of 4.4 MPa, where the circumferential strain reached 1%. The cumulative number of AE events recorded from the wideband piezoelectric and FBG corresponded well as the AE activity increased with higher pressure. When the pressure was held at 3 MPa it was seen that the AE activity decreased for as long as the pressure was maintained constant.

A more sensitive FBG AE device was manufactured with the assistance of a steel tube. One free-end of a strain-free FBG sensor was packaged into a steel tube, which was bonded to a structure. As the FBG was isolated inside the tube, it did not directly interact with the AE waves. One of the fibre ends was bonded to the structure with the AE wave coupled through the fibre into the grating, which provided a more sensitive FBG. The sensor was compared with piezoelectric sensors with a 200 kHz resonance frequency. Tensile loading tests were conducted on an aluminum alloy plate to test the device’s sensitivity to AE data [180].

Additional experiments included the investigation of fatigue crack propagation in stainless steel using an FBG and a conventional piezoelectric sensor as reference [37]. The responses of the FBG and piezoelectric sensors were evaluated with the help of an ultrasonic transmitter driven by a ‘spike’ and ‘tone-burst’ signal, i.e., signals of broadband and narrowband frequency characteristics, respectively. The two different sensors were mounted to a stainless-steel plate with a pre-existing crack starter notch, which resulted in the generation of AE events. The FBG was coated with polyimide before its grating area was secured with strain gauge cement at a pre-determined distance from the crack notch. The ultrasonic transmitter was coupled to the specimen using silicon vacuum grease and secured with adhesive tape. The device was also aligned with the FBG fibre axis. Fatigue tests were carried out with loads ranging from 35 kN to 50 kN. These were stopped and the ultrasonic response measured when the crack reached predetermined lengths under cyclic loading. The Bragg grating sensor was found to have a higher sensitivity to ultrasonic waves along its fibre axis but lower perpendicularly.

The directionality effect on the sensor sensitivity was previously reported in [181], where the amplitude in the parallel direction was one hundred times stronger than that in the perpendicular direction. This meant that the FBG was more susceptible to variations from interference, however. This behaviour in the response of FBGs can be advantageous however in comparison with conventional piezoelectric sensors, whose ultrasonic sensitivity is omnidirectional if the location of the damage source can be predicted. Systems using a broadband light source have the advantage of being low cost but having low sensitivity. One study however, utilised a sensing system with a laser light source that significantly increased the ultrasonic sensitivity. The technique was also adopted in [174] and again in [182], where fatigue cracks in stainless steel were investigated using an FBG ultrasonic sensing system.

Crack detection tests in a metallic material were conducted with conventional piezoelectric sensors and FBGs [182]. The precision of location analysis of crack tips was compared. This reference proposed a mobile FBG sensor that was not restricted to single use. The sensor was glued to an acrylate plate which could then be coupled to any test specimen for multiple use. In this study, the plate was coupled to the specimen with water. The ultrasonic waves travel through the test specimen and propagate across the acrylate plate subsequently interacting with the FBG. The mobile sensor was able to detect ultrasound in any orientation around the fibre just as a conventional piezoelectric sensor. Hence, mobile FBGs were found to be applicable for ultrasonic inspection.

FBGs have also been utilised for hydrophone-related applications. An optical fibre hydrophone was constructed from an FBG based on the intensity modulation of laser light under the influence of sound pressure [183]. The sensor detected sound pressure by the intensity modulation of a narrow spectral bandwidth laser light source. The principle depends on a shift in the reflection or transmission spectrum curve—a result of a sound pressure wave. The study identified a fundamental equation that relates the intensity $\left(I_{in}\right)$ and $\left(\lambda_{in}\right)$ wavelength of the incident light on the FBG to the intensity of the light transmitted through the sensor $\left(I_{t}\right)$. The transmittance of the FBG at the optical wavelength (λ), is given by T(λ) [183]:

(18) $$I_{t}=I_{in}T\left(\lambda_{in}\right)$$

The relationship of the sound pressure dependence on the signal output was investigated. It was concluded that the signal output was proportional to the sound pressure applied to the sensor. Moreover, the FBG hydrophone could be operated across a large frequency range with reasonably flat frequency characteristics. The small localised sensing region meant a minimal sensitivity to external disturbances.

Furthermore, FBG sensors have been used as displacement sensors. An extrinsic fibre optic vibration sensor manufactured from plastic multi-mode fibre was able to monitor a vibration frequency range of 100–3000 Hz, over three data sampling ranges [184]. Plastic FBGs have been praised for their high mechanical flexibility and numerical aperture. In the paper, a silicon photo detector was employed due to its high-speed detection and wide range optical response. It was thus compatible with a broadband light source—a 633 nm visible He-Ne laser. The linearity of the vibration sensor between the output signal and displacement was evaluated as a guidance for vibration measurement of the sensor. The data was taken from the diaphragm of a loudspeaker at zero vibration frequency with a linear range of linearity at around 99%. Moreover, the stability of the sensor’s output demonstrated a low percentage of measurement error at less than 0.37%. The highest sensitivity was recorded with displacements between 0.15 mm and 3.00 mm. The authors highlighted that frequency measurements should be taken within this displacement range in the future.

## 8. Conclusions

The research and development, and application trends of fibre optic sensors for structural health monitoring over the past few decades, have been reviewed and discussed in detail. Past and recent studies investigating optical fibre sensors and their applications have been considered in an effort to present a holistic picture of the current state of the art of the FOAES technology. There has been significantly more research effort spent on FBG AE sensing over interferometric sensing methods due to their simplicity and advantages of multiplexing since wavelength multiplexing is easier to implement than time-domain multiplexing. Fabry-Perot AE sensors, however, have been shown to be the most sensitive devices developed to date. Disadvantages surrounding the Mach-Zehnder and Michelson devices stem from the fact that they require two optical fibres. Though this is overlooked with Fused Tapered Couplers due to the fibre fusion at the sensing region. Whereas, Fabry-Perot and Fibre Bragg Grating devices can be constructed from a single fibre, reducing setup costs, maintenance costs and size of the device. Other multiplexing considerations have considered multi-functional sensors detailing the measurement of multiple parameters simultaneously for strain, refractive index, and temperature. However, only a few studies have considered the inclusion of AE capability in multi-functional fibre optic sensors. Multi-measurand AE sensors with AE capability are likely to increase the interest of the industry in using such sensors over state of the art piezoelectric AE sensors in complex operational environments.

In summary, Table 2 provides a SWOT analysis highlights the main strengths, weaknesses, opportunities, and threats of fibre optic sensing for structural health monitoring.

## Figures and Tables

**Table 1:** An overview of the types of sensors discussed in this review.

Sensor	Monitoring Methods and Techniques	Typical Uses
Fused Tapered Coupler	Merging of two optical fibres via etching, polishing or fibre fusion. An AE stress wave interacts with the fibres causing a change in the effective strain field, thus altering the coupling ratio output of the two fibres.	Acoustic emission, temperature, refractive index
Mach-Zehnder interferometer	A beam splitter separates incoming light into two components to a sensing and reference fibre. The sensing fibre is usually coated with a material that is sensitive to the parameter of interest. The phase of the light propagating through the sensing fibre alters with the influence of an external parameter.	Acoustic emission, strain, refractive index, temperature, pressure, displacement
Michelson interferometer	Like the Mach-Zehnder interferometer, a beam splitter separates incoming light into two components. The sensitivity of the interferometer can be dependent on the length of the exposed fibre.	Acoustic emission, strain, refractive index, temperature, pressure, displacement
Fabry-Perot interferometer	Insertion of two cleaved fibres into a capillary housing. Analysis of the transmitted and reflected waves between the two fibre-ends creates a multiple peak wavelength trace on an optical spectrum analyser.	Acoustic emission, strain, refractive index
Fibre Bragg grating	Manufactured by inscribing a grating into the fibre core with a high-powered UV laser. The reflection of a low-power laser captured by an optical spectrum analyser determines the wavelength peak from the grating.	Acoustic emission, strain, temperature, displacement, and pressure

**Table 2:** A brief SWOT analysis of FOAES.

Strengths	Weaknesses	Opportunities	Threats
Inherent small size and versatility	Reproducibility difficulties during manufacture	Multiplexing of different sensors attached to one fibre	Piezoelectric sensors offer established and reliable sensing systems
Reduced weight	Limited durability when mounting	Available development of automated fabrication process	Limited automated manufacturing available
Immunity to electromagnetic interference	Lower tensile and compressive strength compared with piezoelectric AE sensors	Remote condition monitoring	Some FOS configurations cease to operate in the presence of static strain
Higher operational temperature than piezoelectric sensors	The material cost can often exceed that of some piezoelectric sensors in the long term	Compatibility with telemetry and optical communications	Multiple-use piezoelectric sensors are often preferred over single-use FOAES
Can be embedded into composite structures	High sensitivity limited to along the fibre sensor axis	Collaboration with multiple industries available including aerospace, rail, energy and marine	The increased contact area of piezoelectric sensors to the substrate generally offer increased SNR

## References

[1] Uprety B., Kim S., Mathews V.J., Adams D.O. (2015). A comparative evaluation of piezoelectric sensors for acoustic emission-based impact location estimation and damage classification in composite structures. AIP Conference Proceedings.

[2] Cai J., Qiu L., Yuan S., Shi L., Liu P., Liang D., Hu N. (2012). Structural Health Monitoring for Composite Materials. Composites and Their Applications.

[3] Bockenheimer C., Speckmann H., Team I. Validation, verification and implementation of SHM at Airbus. Proceedings of the 9th International Workshop on Structural Health Monitoring (IWSHM 2013).

[4] Di Sante R. (2015). Fibre Optic Sensors for Structural Health Monitoring of Aircraft Composite Structures: Recent Advances and Applications. Sensors.

[5] Kister G., Winter D., Badcock R.A., Gebremichael Y.M., Boyle W.J.O., Meggitt B.T., Grattan K.T.V., Fernando G.F. (2007). Structural health monitoring of a composite bridge using Bragg grating sensors. Part. 1: Evaluation of adhesives and protection systems for the optical sensors. Eng. Struct..

[6] Alani A.M., Aboutalebi M., Kilic G. (2014). Integrated health assessment strategy using NDT for reinforced concrete bridges. NDT E Int..

[7] Maria M. (2013). Advanced composite materials of the future in aerospace industry. INCAS Bull..

[8] Drewry M., Georgiou G. (2007). A review of NDT techniques for wind turbines. Insight.

[9] Galappaththi U.I.K., de Silva A.K.M., Macdonald M., Adewale O.R. (2012). Review of inspection and quality control techniques for composite wind turbine blades. Insight Non Destr. Test. Cond. Monit..

[10] Camacho J., Atehortua D., Cruza J.F., Brizuela J., Ealo J. (2018). Ultrasonic crack evaluation by phase coherence processing and TFM and its application to online monitoring in fatigue tests. NDT E Int..

[11] Ciampa F., Mahmoodi P., Pinto F., Meo M. (2018). Recent advances in active infrared thermography for non-destructive testing of aerospace components. Sensors.

[12] Hung Y.Y. (1989). Shearography: A novel and practical approach for nondestructive inspection. J. Nondestr. Eval..

[13] Hellier C., Hellier C. (2012). Radiographic Testing. Handbook of Nondestructive Evaluation.

[14] Munoz V., Vales B., Perrin M., Pastor M., Welemane H., Cantarel A., Karama M. (2016). Damage detection in CFRP by coupling acoustic emission and infrared thermography. Compos. Part B Eng..

[15] Sagaidak A., Bardakov V., Elizarov S., Terentyev D. (2015). The Use of Acoustic Emission Method in the Modern Construction.

[16] Hellier C. (2012). Acoustic Emission Testing. Handbook of Nondestructive Evaluation.

[17] Sachse W., Yamaguchi K., Roget J. (1991). Acoustic Emission: Current Practice and Future Directions.

[18] Chandarana N., Sanchez D.M., Soutis C., Gresil M. (2017). Early Damage Detection in Composites during Fabrication and Mechanical Testing. Materials.

[19] Crawford A., Droubi M.G., Faisal N.H. (2018). Analysis of acoustic emission propagation in metal-to-metal adhesively bonded joints. J. Nondestr. Eval..

[20] Diakhate M., Angellier N., Pitti R.M., Dubois F. (2017). On the crack tip propagation monitoring within wood material: Cluster analysis of acoustic emission data compared with numerical modelling. Constr. Build. Mater..

[21] Fedele R., Pratico F.G., Carotenuto R., della Corte F.G. Damage detection into road pavement through acoustic signature analysis: First results. Proceedings of the 24th International Congress on Sound and Vibration (ICSV 24).

[22] Mujica L., Rodellar J., Vehí J. (2013). A review of impact damage detection in structures using strain data. Int. J. COMADEM.

[23] Doyle C.T.M., Chen R., Liu T., Zheng G., Fernando G.F. Fiber optic acoustic emission sensor based on a fused tapered coupler. Proceedings of the SPIE’s 9th Annual International Symposium on Smart Structures and Materials.

[24] Chen R., Liao Y., Zheng G., Fernando G.F. (2004). A novel ultrasound fibre optic sensor based on a fused-tapered optical fibre coupler. Meas. Sci. Technol..

[25] Fornel F.d., Ragdale C.M., Mears R.J. (1984). Analysis of Single-Mode Fused Tapered Fibre Couplers. Microwaves Opt. Antennas IEE Proc. H.

[26] Pierce S.G., Philp W.R., Gachagan A., McNab A., Hayward G., Culshaw B. (1996). Surface-bonded and embedded optical fibers as ultrasonic sensors. Appl. Opt..

[27] Lan C., Zhou W., Xie Y. (2018). Detection of Ultrasonic Stress Waves in Structures Using 3D Shaped Optic Fiber Based on a Mach.-Zehnder Interferometer. Sensors.

[28] Tada K., Yuki H. (2017). detection of acoustic emission signals with the fabry-perot interferometer type optical fiber sensor. J. Acoust. Emiss..

[29] Islam M., Ali M.M., Lai M., Lim K., Ahmad H. (2014). Chronology of Fabry-Perot Interferometer Fiber-Optic Sensors and Their Applications: A Review. Sensors.

[30] Grouve W.J.B., Warnet L., de Boer A., Akkerman R., Vlekken J. (2008). Delamination detection with fibre Bragg gratings based on dynamic behaviour. Compos. Sci. Technol..

[31] Wild G., Hinckley S. Fiber Bragg Grating Sensors for Acoustic Emission and Transmission Detection Applied to Robotic NDE in Structural Health Monitoring. Proceedings of the 2007 IEEE Sensors Applications Symposium.

[32] Guo N., Cawley P. (1993). Lamb wave propagation in composite laminates and its relationship with acousto-ultrasonics. NDT E Int..

[33] Wang D., Ye L., Tang Y., Lu Y. (2012). Monitoring of delamination onset and growth during Mode I and Mode II interlaminar fracture tests using guided waves. Compos. Sci. Technol..

[34] Sohn H., Park G., Wait J.R., Limback N.P., Farrar C.R. (2003). Wavelet-based active sensing for delamination detection in composite structures. Smart Mater. Struct..

[35] Gangadharan R., Prasanna G., Bhat M.R., Murthy C.R.L., Gopalakrishnan S. (2009). Acoustic emission source location and damage detection in a metallic structure using a graph-theory-based geodesic approach. Smart Mater. Struct..

[36] Sherafat M.H., Quaegebeur N., Hubert P., Lessard L., Masson P. (2016). Finite element modeling of Lamb wave propagation in composite stepped joints. J. Reinf. Plast. Compos..

[37] Tsuda H., Lee J.-R., Guan Y. (2006). Fatigue crack propagation monitoring of stainless steel using fiber Bragg grating ultrasound sensors. Smart Mater. Struct..

[38] Cawley P., Alleyne D. (1996). The use of Lamb waves for the long range inspection of large structures. Ultrasonics.

[39] Eckert E.G., Maresca J.W., Hillger R.W., Yezzi J.J., Durgin P., Young T. (1992). Location of Leaks in Pressurized Petroleum Pipelines by Means of Passive-Acoustic Sensing Methods. Leak Detection for Underground Storage Tanks.

[40] Mukhopadhyay C.K., Haneef T.K., Rao B.P.C., Jayakumar T. (2014). On-line Monitoring of Engineering Components Using Acoustic Emission Technique. Procedia Eng..

[41] Lacidogna G., Manuello A., Niccolini G., Accornero F., Carpinteri A., Ohtsu M. (2015). Acoustic emission wireless monitoring of structures. Acoustic Emission and Related Non-Destructive Evaluation Techniques in the Fracture Mechanics of Concrete.

[42] Masmoudi S., el Mahi A., Turki S. (2015). Use of piezoelectric as acoustic emission sensor for in situ monitoring of composite structures. Compos. Part B Eng..

[43] Yu F., Okabe Y., Wu Q., Shigeta N. (2016). Fiber-optic sensor-based remote acoustic emission measurement of composites. Smart Mater. Struct..

[44] Shiotani T., Yuyama S., Carlos M., Vahaviolos S.J. (2000). Continuous monitoring of rock failure by a remote AE system. J. Acoust. Emiss..

[45] Pang D., Sui Q., Wang M., Guo D., Sai Y. (2018). Development of high temperature acoustic emission sensing system using fiber Bragg grating. Photonic Sens..

[46] Yu F., Okabe Y. (2017). Fiber-optic sensor-based remote acoustic emission measurement in a 1000 °C environment. Sensors.

[47] Leal-Junior A., Frizera-Neto A., Marques C., Pontes M.J. (2018). A polymer optical fiber temperature sensor based on material features. Sensors.

[48] Broadway C., Kalli K., Theodosiou A., Zubel M., Sugden K., Megret P., Caucheteur C. L-band CYTOP Bragg gratings for ultrasound sensing. Proceedings of the Micro-Structured and Specialty Optical Fibres V.

[49] Bucaro J.A., Dardy H.D., Carome E.F. (1977). Fiber-optic hydrophone. J. Acoust. Soc. Am..

[50] Kawasaki B.S., Hill K.O., Lamont R.G. (1981). Biconical-taper single-mode fiber coupler. Opt. Lett..

[51] Sorazu B., Thursby G., Culshaw B., Dong F., Pierce S.G., Yang Y., Betz D. (2003). Optical generation and detection of ultrasound. Strain.

[52] Dewhurst R.J., Edwards C.E., McKie A.D.W., Palmer S.B. (1987). Comparative study of wide-band ultrasonic transducers. Ultrasonics.

[53] Mistras WD Sensor (Wideband Differential Sensor).

[54] Wild G., Hinckley S. (2008). Acousto-Ultrasonic Optical Fiber Sensors: Overview and State-of-the-Art. IEEE Sens. J..

[55] Hromadka J., Korposh S., Partridge M.C., James S.W., Davis F., Crump D., Tatam R.P. (2017). Multi-parameter measurements using optical fibre long period gratings for indoor air quality monitoring. Sens. Actuators B Chem..

[56] Nair A.K., Machavaram V.R., Mahendran R.S., Pandita S.D., Paget C., Barrow C., Fernando G.F. (2015). Process. monitoring of fibre reinforced composites using a multi-measurand fibre-optic sensor. Sens. Actuators B Chem..

[57] Thursby G., Culshaw B., Betz D.C. (2008). Multifunctional fibre optic sensors monitoring strain and ultrasound. Fatigue Fract. Eng. Mater. Struct..

[58] Rao Y.J., Henderson P.J., Jackson D.A., Zhang L., Bennion I. (1997). Simultaneous strain, temperature and vibration measurement using a multiplexed in-fibre-Bragg-grating/fibre-Fabry-Perot sensor system. Electron. Lett..

[59] Oliveira R., Osório J.H., Aristilde S., Bilro L., Nogueira R.N., Cordeiro C.M.B. (2016). Simultaneous measurement of strain, temperature and refractive index based on multimode interference, fiber tapering and fiber Bragg gratings. Meas. Sci. Technol..

[60] Jones M. (2008). Structural-health monitoring: A sensitive issue. Nat. Photonics.

[61] Wevers M., Rippert L., Papy J.M., van Huffel S., Hemelrijck D., Anastaopoulos A., Melanitis N.E. Damage in CFRP composite materials monitored with intensity modulated fiber optic sensors. Emerging Technologies in NDT, Proceedings of the 3rd International Conference on Emerging Technologies in Non-Destructive Testing, Thessaloniki, Greece, 26–28 May 2003.

[62] Wei P., Han X., Xia D., Liu T., Lang H. (2018). Novel fiber-optic ring acoustic emission sensor. Sensors.

[63] Cole J.H., Danver B.A., Bucaro J.A. (1982). Synthetic-Heterodyne Interferometric Demodulation. IEEE Trans. Microwav. Theory Tech..

[64] Paschotta R. Cladding Modes. Encyclopedia of Laser Physics and Technology October 2008. https://www.rp-photonics.com/cladding_modes.html.

[65] Ivanov O.V., Nikitov S.A., Gulyaev Y.V. (2006). Cladding modes of optical fibers: Properties and applications. Physics-Uspekhi.

[66] Haggans C.W., Singh H., Varner W.F., Wang J. (1998). Narrow-depressed cladding fiber design for minimization of cladding mode losses in azimuthally asymmetric fiber Bragg gratings. J. Lightwave Technol..

[67] Berendt M.O., Gruner-Nielsen L., Bjarklev A., Soccorich C.E. (1999). Reduction of cladding mode coupling losses in fiber Bragg gratings. 1999 SBMO/IEEE MTT-S International Microwave and Optoelectronics Conference.

[68] Weik M. (2012). Communications Standard Dictionary.

[69] Leon-Saval S.G., Fontaine N.K., Amezcua-Correa R. The photonic lantern. Proceedings of the 2014 Conference on Lasers and Electro-Optics (CLEO)—Laser Science to Photonic Applications.

[70] (2020). 630HP—Single Mode Optical Fiber, 600–770 nm, Ø125 µm Cladding.

[71] (2020). Hollow Round Glass Capillaries ID 0.60 mm OD 0.84 mm (75 capillaries per pack).

[72] Grosse C.U., Grosse C., Ohtsu M. (2008). Wireless Sensing and Acoustic Emission Array Techniques. Acoustic Emission Testing: Basics for Research—Applications in Civil. Engineering.

[73] Wang L., Liu Y., Fu W., Li F., Zhao Z., Yu K. (2017). Source location using an optimized microfiber coupler sensor based on modal acoustic emission method. Struct. Control Health Monit..

[74] Rosiek M., Martowicz A., Uhl T. An Overview of Electromechanical Impedance Method for Damage Detection in Mechanical Structures. Proceedings of the 6th European Workshop on Structural Health Monitoring.

[75] Hellier C. (2012). Introduction to Nondestructive Testing. Handbook of Nondestructive Evaluation.

[76] Williams R.V. (1980). Acoustic Emission.

[77] Zhou C., Zhang Y. (2012). Particle filter based noise removal method for acoustic emission signals. Mech. Syst. Signal Process..

[78] Corporation P.A. (2007). PCI-2 Based AE System User’s Manual Rev 3.

[79] Sun L., Li Y. Acoustic emission sound source localization for crack in the pipeline. Proceedings of the 2010 Chinese Control and Decision Conference.

[80] Fasana A., Garibaldi L. (2007). Measurement of Acoustic Emission Signals: Influence of the Couplant.

[81] Hamstad M.A. (2009). Some observations on rayleigh waves and acoustic emission in thick steel plates. J. Acoust. Emiss..

[82] Fu T., Li Q., Liu Y., Leng J. (2009). A novel embedded fiber optic acoustic emission sensor and its applications for monitoring failures of composite laminates. Smart Sensor Phenomena, Technology, Networks, and Systems 2009.

[83] Hughes J.M., Vidler J., Ng C., Khanna A., Mohabuth M., Rose L.R.F., Kotousov A. (2018). Comparative evaluation of in situ stress monitoring with Rayleigh waves. Struct. Health Monit..

[84] Chen R., Bradshaw T., Burns J., Cole P., Jarman P., Pedder D., Theobald R., Fernando G.F. (2006). Linear location of acoustic emission using a pair of novel fibre optic sensors. Meas. Sci. Technol..

[85] Unnþórsson R.N., Sikorski D.W. (2013). Hit Detection and Determination in AE Bursts. Acoustic Emission—Research and Applications.

[86] Ogura G. (2001). Laser stripping of optical fibers opens up new applications. Laser Focus World.

[87] Zlatanov N. Introduction to Fiber Optics Theory. https://www.academia.edu/28851433/Introduction_to_Fiber_Optics_Theory.

[88] Son G., Jung Y., Yu K. (2017). Tapered Optical Fiber Couplers Fabricated by Droplet-Based Chemical Etching. IEEE Photonics J..

[89] Zhang H., Healy N., Dasgupta S., Hayes J.R., Petrovich M.N., Richardson D.J., Peacock A.C. (2017). A Tuneable Multi-Core to Single Mode Fiber Coupler. IEEE Photonics Technol. Lett..

[90] Pal B. (2003). Fabrication and modeling of fused biconical tapered fiber couplers. Fiber Integr. Opt..

[91] Zhang J.-L., Mao Z.-M., Lin Z.-Q. (1989). Measurements and analyses of fields in fused tapered single-mode fiber couplers. Appl. Opt..

[92] Lee B.H., Eom J.B., Kim J., Moon D.S., Paek U.C., Yang G.H. (2002). Photonic crystal fiber coupler. Opt. Lett..

[93] Bogonez F.D.N. (2017). Manufacturing and Characterisation of a Fibre Optic Acoustic Emission Sensor. School of Metallurgy and Materials.

[94] Burns J.M. (2011). Development and Characterisation of a Fibre-Optic Acoustic Emission Sensor. School of Metallurgy and Materials.

[95] Snyder A.W. (1972). Coupled-Mode Theory for Optical Fibers. J. Opt. Soc. Am..

[96] Shuai C.-j., Duan J.-A., Zhong J. (2006). Novel manufacturing method of optical fiber coupler. J. Cent. South Univ. Technol..

[97] Eisenmann M., Weidel E. (1988). Single-mode fused biconical couplers for wavelength division multiplexing with channel spacing between 100 and 300 nm. J. Lightwave Technol..

[98] Birks T.A., Russell P.S.J., Culverhouse D.O. (1996). The acousto-optic effect in single-mode fiber tapers and couplers. J. Lightwave Technol..

[99] Pidishety S. Fused Fiber Couplers: Basic Theory and Automated Fabrication. https://www.semanticscholar.org/paper/Fused-Fiber-Couplers%3A-Basic-Theory-and-Automated/ccd28657365d8e9f66a6e008b677938760999dc1.

[100] Matthews A.L., Murphy K.A., Rogers R.E., Claus R.O. Acoustic Fiber Waveguide Coupler. Proceedings of the IEEE 1987 Ultrasonics Symposium.

[101] Ghatak A., Thyagarajan K. An. (1998). Introduction to Fiber Optics.

[102] Ghatak A., Thyagarajan K., Ghatak A., Thyagarajan K. (1998). Single-mode fiber optic components. An Introduction to Fiber Optics.

[103] Li Y., Wang X., Bao X. (2011). Sensitive acoustic vibration sensor using single-mode fiber tapers. Appl. Opt..

[104] Musa B., Kamil Y.M., Bakar M.H., Noor A.S.M., Ismail A., Mahdi M. (2016). Investigating the effect of taper length on sensitivity of the tapered-fiber based temperature sensor. Mater. Sci..

[105] Chen R., Zheng G., Liu T., Fernando G.F., Liao Y.B. (2000). A Novel Acoustic Emission Fiber Optic Sensor Based on a Single Mode Optical Fiber Coupler. Chin. J. Lasers.

[106] Li F., Liu Y., Wang L., Zhao Z. (2015). Investigation on the response of fused taper couplers to ultrasonic wave. Appl. Opt..

[107] Wang S., Lu P., Zhang L., Liu D., Zhang J. (2014). Optical fiber acoustic sensor based on nonstandard fused coupler and aluminum foil. IEEE Sens. J..

[108] Xu J., Ma B., Zhou X. Theoretical analysis and experimental investigation of fiber-optic coupler’s strain characteristic. Proceedings of the 2009 IEEE International Conference on Automation and Logistics.

[109] Butler. T., Krishnamurthy S., Badcock R., Chen R., Chang F. (2003). A Low-Cost Fiber Optic Acoustic Emission Sensor for Damage Detection in Engineering Composite Materials and Structures. Structural Health Monitoring 2003: From Diagnostics & Prognostics to Structural Health Management, Proceedings of the 4th International Workshop on Structural Health Monitoring, Stanford University Stanford, CA, USA, 15–17 September 2003.

[110] Badcock R., Krishnamurthy S., Fernando G.F., Butler T., Chen R., Tetlow J. (2004). Health monitoring of composite structures using a novel fibre optic acoustic emission sensors. Proceedings of the 11th European Conference on Composite Materials.

[111] Birks T.A. (1988). Practical tuning mechanism for fused-tapered couplers. Opt. Lett..

[112] Fu T., Liu Y., Li Q., Leng J. (2009). Fiber optic acoustic emission sensor and its applications in the structural health monitoring of CFRP materials. Opt. Lasers Eng..

[113] Fu T., Liu Y., Lau K., Leng J. (2014). Impact source identification in a carbon fiber reinforced polymer plate by using embedded fiber optic acoustic emission sensors. Compos. Part B Eng..

[114] Thursby G., Culshaw B. Evaluation of the internal strains and stresses produced in a plate by propagating Lamb waves through the use of fibre optic sensors. Proceedings of the SPIE Smart Structures and Materials + Nondestructive Evaluation and Health Monitoring.

[115] Papasalouros D., Tsopelas N., Anastasopoulos A., Kourousis D., Lekou D.J., Mouzakis F. (2013). Acoustic emission monitoring of composite blade of NM48/750 NEG-MICON wind turbine. J. Acoust. Emiss..

[116] Beattie A.G. (1997). Acoustic Emission Monitoring of a Wind Turbine Blade during a Fatigue Test.

[117] Sutherland H., Beattie A., Hansche B., Musial W., Allread J., Johnson J., Summers M. (1994). The Application of Non-Destructive Techniques to the Testing of a Wind Turbine Blade.

[118] Joosse P.A., Blanch M.J., Dutton A.G., Kouroussis D.A., Philippidis T.P., Vionis P.S. (2002). Acoustic Emission Monitoring of Small Wind Turbine Blades. J. Sol. Energy Eng..

[119] Doyle C., Tuck C., Chen R., Fernando G.F., Zheng G., Liu T., Joosse P., Van Delft V., Dutton A., Blanch M. (2003). Application of a fibre-optic acoustic emission sensor to the fatigue testing of wind turbine blades. https://www.researchgate.net/publication/333517246_Application_of_a_fibre-optic_acoustic_emission_sensor_to_the_fatigue_testing_of_wind_turbine_blades.

[120] Her S.-C., Yang C.-M. (2012). Dynamic strain measured by mach.-zehnder interferometric optical fiber sensors. Sensors.

[121] Heijmans J.A.C., Cheng L.K., Wieringa F.P. (2009). Optical Fiber Sensors for Medical Applications—Practical Engineering Considerations.

[122] Ahsani V., Ahmed F., Jun M.B.G., Bradley C. (2019). Tapered fiber-optic mach-zehnder interferometer for ultra-high. sensitivity measurement of refractive index. Sensors.

[123] Gao S., Ji C., Ning Q., Chen W., Li J. (2020). High-sensitive Mach.-Zehnder interferometric temperature fiber-optic sensor based on core-offset splicing technique. Opt. Fiber Technol..

[124] Liu Y., Lin H., Dai Y., Zhou A., Yuan L. (2018). Fiber In-Line Mach–Zehnder Interferometer for Gas. Pressure Sensing. IEEE Sens. J..

[125] Shen C., Wang Y., Chu J., Lu Y., Li Y., Dong X. (2014). Optical fiber axial micro-displacement sensor based on Mach.-Zehnder interferometer. Opt. Express.

[126] Bucaro J.A., Dardy H.D., Carome E.F. (1977). Optical fiber acoustic sensor. Appl. Opt..

[127] Xu Y. (2014). Delamination detection at web/flange junction of I-section composite beam with fiber optical interferometer sensor. Compos. Part B Eng..

[128] Hocker G.B. (1979). Fiber optic acoustic sensors with composite structure: An analysis. Appl. Opt..

[129] Hocker G.B. (1979). Fiber-optic acoustic sensors with increased sensitivity by use of composite structures. Opt. Lett..

[130] Lagakos N., Hickman T.R., Ehrenfeuchter P., Bucaro J.A., Dandridge A. (1990). Planar flexible fiber-optic acoustic sensors. J. Lightwave Technol..

[131] Liang S., Zhang C., Lin W., Li L., Li C., Feng X., Lin B. (2009). Fiber-optic intrinsic distributed acoustic emission sensor for large structure health monitoring. Opt. Lett..

[132] Gong J., MacAlpine J.M.K., Jin W., Liao Y. (2001). Locating acoustic emission with an amplitude-multiplexed acoustic sensor array based on a modified Mach–Zehnder interferometer. Appl. Opt..

[133] Zhu W., Li D., Liu J., Wang R. (2020). Membrane-free acoustic sensing based on an optical fiber Mach–Zehnder interferometer. Appl. Opt..

[134] Teixeira J.G.V., Leite I.T., Silva S., Frazão O. (2014). Advanced fiber-optic acoustic sensors. Photonic Sens..

[135] Brandenburg. A., Hinkov V., Konz. W., Wagner E., Dandliker R., Spenner K. (1992). Integrated Optic Sensors. Sensors, A Comprehensive Survey.

[136] Tsuda H., Ikeguchi T., Takahashi J., Kemmochi K. (1998). Damage monitoring of carbon-fibre-reinforced plastics using Michelson interferometric fibre-optic sensors. J. Mater. Sci. Lett..

[137] Liu K., Ferguson S.M., Measures R.M. (1990). Fiber-optic interferometric sensor for the detection of acoustic emission within composite materials. Opt. Lett..

[138] Pierce S.G., Culshaw B., Philp W.R., Lecuyer F., Farlow R. (1997). Broadband Lamb wave measurements in aluminium and carbon/glass fibre reinforced composite materials using non-contacting laser generation and detection. Ultrasonics.

[139] Zhang T., Pang F., Liu H., Cheng J., Lv L., Zhang X., Chen N., Wang T. (2016). A fiber-optic sensor for acoustic emission detection in a high. voltage cable system. Sensors.

[140] Lű T., Li Z., Xia D., He K., Zhang G. (2009). Asymmetric Fabry–Pérot fiber-optic pressure sensor for liquid-level measurement. Rev. Sci. Instrum..

[141] Zhao J.H., Shi Y.K., Shan N., Yuan X.Q. (2008). Stabilized fiber-optic extrinsic Fabry–Perot sensor system for acoustic emission measurement. Opt. Laser Technol..

[142] Yu B., Kim D.W., Deng J., Xiao H., Wang A. (2003). Fiber Fabry-Perot sensors for detection of partial discharges in power transformers. Appl. Opt..

[143] Dong Y., Karbhari V.M. (2013). Non-destructive evaluation (NDE) of composites: Using fiber optic sensors. Non-Destructive Evaluation (NDE) of Polymer Matrix Composites.

[144] Wang Z., Shen F., Song L., Wang X., Wang A. (2007). Multiplexed fiber fabry-perot interferometer sensors based on ultrashort bragg gratings. IEEE Photonics Technol. Lett..

[145] Kbashi H.J. (2012). Fabrication of Submicron-Diameter and Taper Fibers Using Chemical Etching. J. Mater. Sci. Technol..

[146] Read I., Foote P., Murray S. (2001). Optical fibre acoustic emission sensor for damage detection in carbon fibre composite structures. Meas. Sci. Technol..

[147] Bucaro J.A., Carome E.F. (1978). Single fiber interferometric acoustic sensor. Appl. Opt..

[148] Tran T.A., Miller W.V., Murphy K.A., Vengsarkar A.M., Claus R.O. (1992). Stabilized extrinsic fiber-optic Fizeau sensor for surface acoustic wave detection. J. Lightwave Technol..

[149] Beard P.C., Mills T.N. (1996). Extrinsic optical-fiber ultrasound sensor using a thin polymer film as a low-finesse Fabry–Perot interferometer. Appl. Opt..

[150] Kim D.-H., Koo B.Y., Kim C.G., Hong C.S. (2004). Damage detection of composite structures using a stabilized extrinsic Fabry–Perot interferometric sensor system. Smart Mater. Struct..

[151] Zhang Q., Zhu Y., Luo X., Liu G., Han M. (2017). Acoustic emission sensor system using a chirped fiber-Bragg-grating Fabry–Perot interferometer and smart feedback control. Opt. Lett..

[152] Pappu R.P. (2012). Acoustic Emission Detection Using Optical Fibre Sensors for Aerospace Applications. Ph.D. Thesis.

[153] Campanella C.E., Cuccovillo A., Campanella C., Yurt A., Passaro V. (2018). Fibre Bragg Grating Based Strain Sensors: Review of Technology and Applications. Sensors.

[154] Majumder M., Gangopadhyay T.K., Chakraborty A.K., Dasgupta K., Bhattacharya D. (2008). K Fibre Bragg gratings in structural health monitoring—Present status and applications. Sens. Actuators A Phys..

[155] Kashyap R., Kashyap R. (2010). Chapter 10—Principles of Optical Fiber Grating Sensors. Fiber Bragg Gratings.

[156] Huang D.W., Liu W.F., Wu C.W., Yang C.C. (2000). Reflectivity-tunable fiber Bragg grating reflectors. IEEE Photonics Technol. Lett..

[157] Liu W.F., Russell P.S.J., Dong L. (1998). 100% efficient narrow-band acoustooptic tunable reflector using fiber Bragg grating. J. Lightwave Technol..

[158] Il D.Y., Su P.H., Yoon K.B. (2004). Tunable narrow-bandwidth optical filter based on acoustically modulated fiber Bragg grating. IEEE Photonics Technol. Lett..

[159] Guo H., Xiao G., Mrad N., Yao J. (2011). Fiber optic sensors for structural health monitoring of air platforms. Sensors.

[160] Minardo A., Cusano A., Bernini R., Zeni L., Giordano M. (2005). Response of fiber Bragg gratings to longitudinal ultrasonic waves. IEEE Trans. Ultrason. Ferroelectr. Freq. Control.

[161] Luo Z., Ye C., Cai Z., Dai X., Kang Y., Xu H. (2007). Numerical analysis and optimization of optical spectral characteristics of fiber Bragg gratings modulated by a transverse acoustic wave. Appl. Opt..

[162] Erdogan T. (1997). Cladding-mode resonances in short- and long-period fiber grating filters. J. Opt. Soc. Am..

[163] Lee R.J., Tsuda H., Koo B.-Y. (2007). Single-mode fibre optic Bragg grating sensing on the base of birefringence in surface-mounting and embedding applications. Opt. Laser Technol..

[164] Rao Y.J. (1999). Recent progress in applications of in-fibre Bragg grating sensors. Opt. Lasers Eng..

[165] James S.W., Korposh S., Lee S.W., Tatam R.P. (2014). A long period grating-based chemical sensor insensitive to the influence of interfering parameters. Opt. Express.

[166] Giurgiutiu V., Giurgiutiu V. (2016). Chapter 7—Fiber-Optic Sensors. Structural Health Monitoring of Aerospace Composites.

[167] Kawasaki B.S., Hill K.O., Johnson D.C., Fujii Y.J.O.L. (1978). Narrow-band Bragg reflectors in optical fibers. Opt. Lett..

[168] Beukema R.P. Embedding technologies of FBG sensors in composites: Technologies, applications and practical use. Proceedings of the 6th European Workshop—Structural Health Monitoring 2012, EWSHM 2012.

[169] Kister G., Winter D., Gebremichael Y.M., Leighton J., Badcock R.A., Tester P.D., Krishnamurthy S., Boyle W.J.O., Grattan K.T.V., Fernando G.F. (2007). Methodology and integrity monitoring of foundation concrete piles using Bragg grating optical fibre sensors. Eng. Struct..

[170] Čápová K., Velebil L., Včelák J. (2020). Laboratory and In-Situ Testing of Integrated FBG Sensors for SHM for Concrete and Timber Structures. Sensors.

[171] Ngoi B.K.A., Paul J., Zhao L.P., Fang Z.P. (2004). Enhanced lateral pressure tuning of fiber Bragg gratings by polymer packaging. Opt. Commun..

[172] Yu F.M., Okabe Y., Wu Q., Shigeta N. (2016). A novel method of identifying damage types in carbon fiber-reinforced plastic cross-ply laminates based on acoustic emission detection using a fiber-optic sensor. Compos. Sci. Technol..

[173] Azmi A.I., Peng G.D. (2013). Failure monitoring of E-glass/vinylester composites using fiber grating acoustic sensor. Photonic Sens..

[174] Tsuda H. (2006). Ultrasound and damage detection in CFRP using fiber Bragg grating sensors. Compos. Sci. Technol..

[175] Tsuda H., Toyama N., Takatsubo J. (2004). Damage detection of CFRP using fiber Bragg gratings. J. Mater. Sci..

[176] Takeda N., Okabe Y., Kuwahara J., Kojima S., Ogisu T. (2005). Development of smart composite structures with small-diameter fiber Bragg grating sensors for damage detection: Quantitative evaluation of delamination length in CFRP laminates using Lamb wave sensing. Compos. Sci. Technol..

[177] Zhou Z., Liu W., Huang Y., Wang H., Jianping H., Huang M., Jinping O. (2012). Optical fiber Bragg grating sensor assembly for 3D strain monitoring and its case study in highway pavement. Mech. Syst. Signal Process..

[178] Betz D.C., Thursby G., Culshaw B., Staszewski W.J. (2006). Identification of structural damage using multifunctional Bragg grating sensors: I. Theory and implementation. Smart Mater. Struct..

[179] Tsuda H., Sato E., Nakajima T., Nakamura H., Arakawa T., Shiono H., Minato M., Kurabayashi H., Sato A. (2009). Acoustic emission measurement using a strain-insensitive fiber Bragg grating sensor under varying load conditions. Opt. Lett..

[180] Lee J.-R., Tsuda H. (2006). Acousto-ultrasonic sensing using capsular fibre Bragg gratings for temperature compensation. Meas. Sci. Technol..

[181] Betz D.C., Thursby G., Culshaw B., Staszewski W.J. (2003). Acousto-ultrasonic sensing using fiber Bragg gratings. Smart Mater. Struct..

[182] Tsuda H., Lee J.R., Guan Y., Takatsubo J. (2007). Investigation of fatigue crack in stainless steel using a mobile fiber Bragg grating ultrasonic sensor. Opt. Fiber Technol..

[183] Takahashi N., Yoshimura K., Takahashi S., Imamura K. (2000). Development of an optical fiber hydrophone with fiber Bragg grating. Ultrasonics.

[184] Abdullah M., Bidin N., Yasin M. (2016). Fiber optic vibration sensor using bifurcated plastic optical fiber. AIP Conf. Proc..

